# Supplementary figures and images for: Genome-wide analysis of R2R3-MYB transcription factors in Japanese morning glory
Source: PLoS One. 2022 Oct 20;17(10):e0271012. doi: 10.1371/journal.pone.0271012 (PMC9584510; doi:10.1371/journal.pone.0271012)

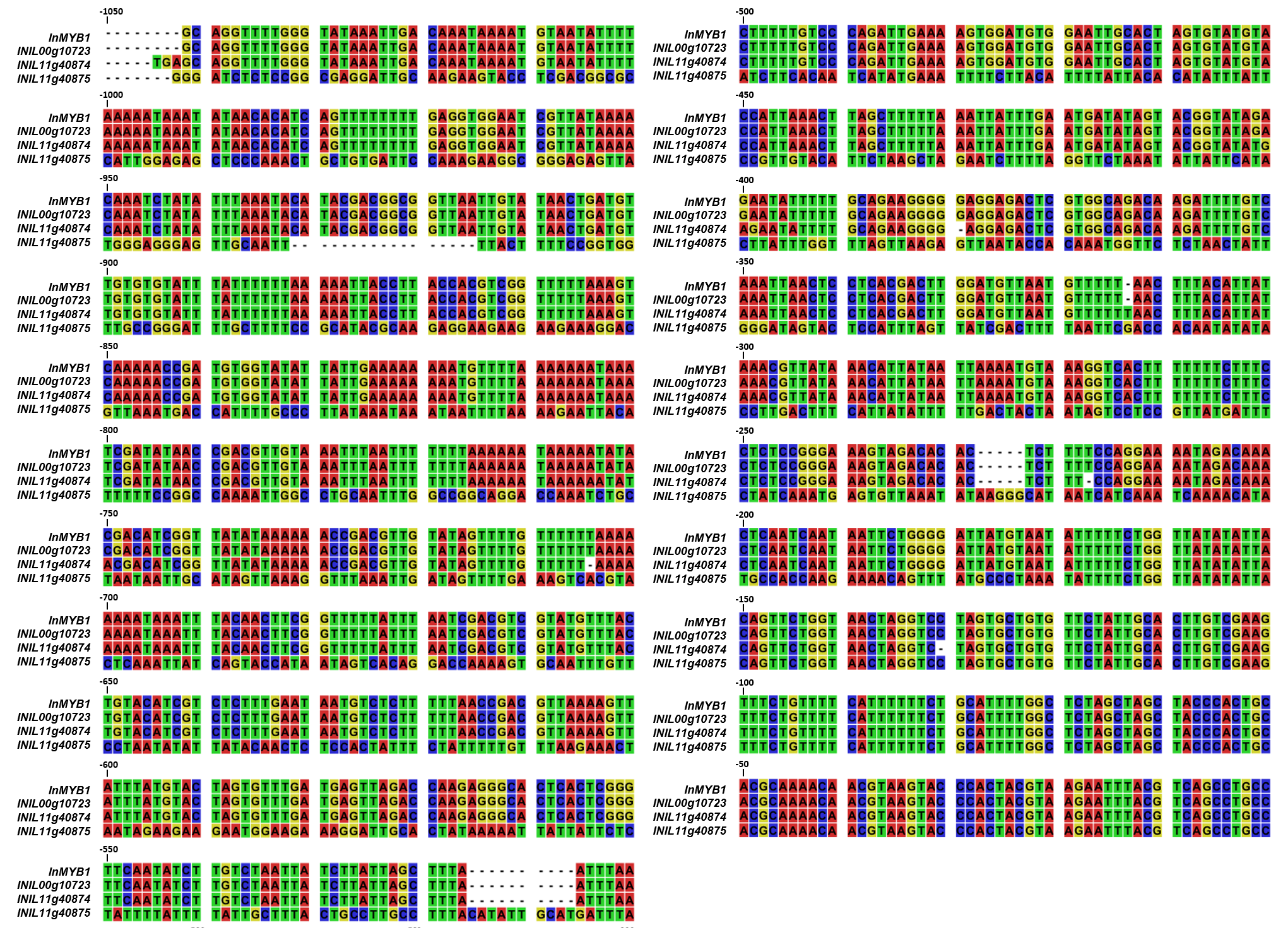

Supplement: S1 Fig — The 1026-bp upstream sequences of INIL00g17023, INIL11g40874 and INIL11g40875 from the transcription start site were aligned. The number above the alignment indicates the position from the transcription start site. (TIF) [file pone.0271012.s001.tif]

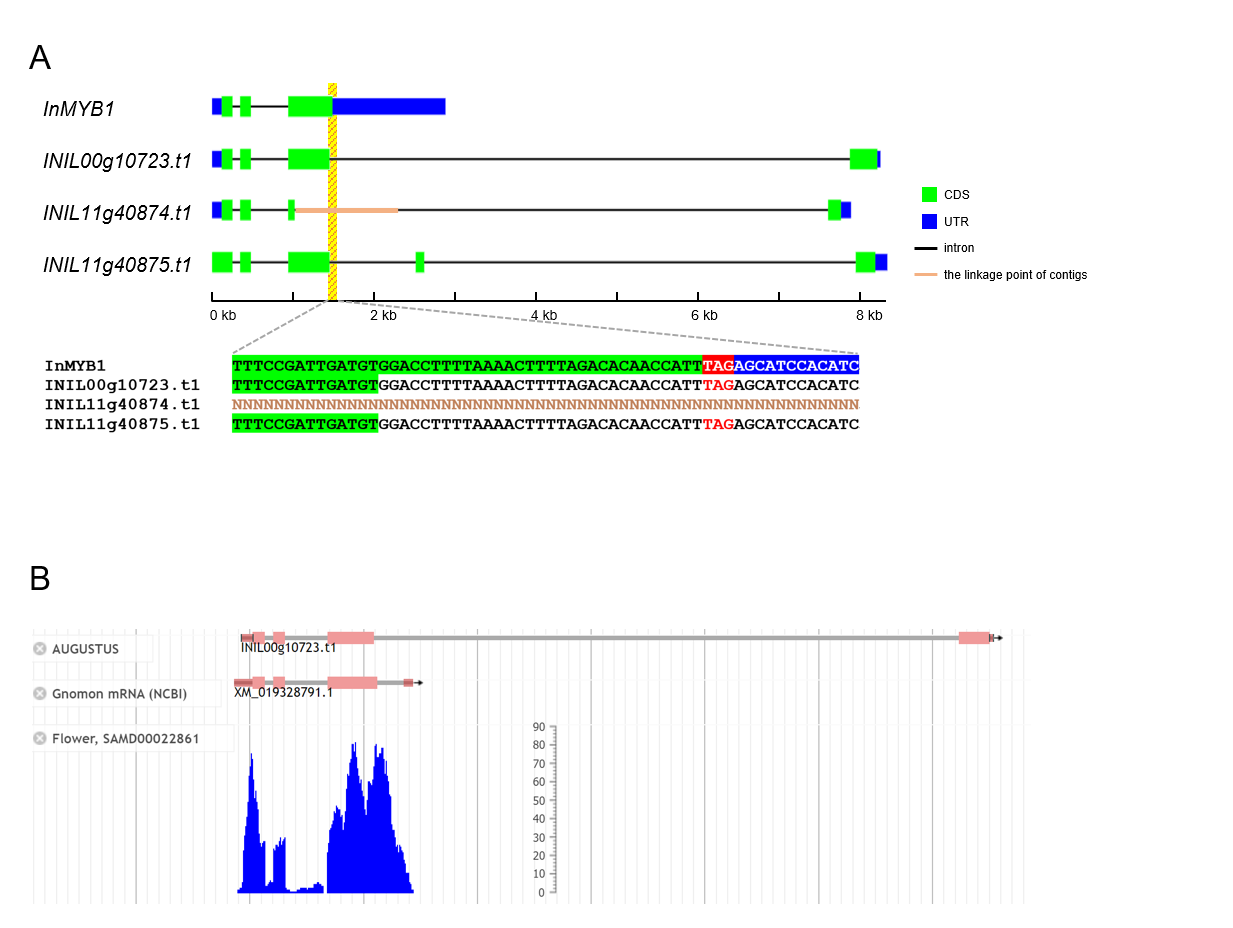

Supplement: S2 Fig — A: INIL00g10723 and INIL11g40875 have stop codons in the same position as InMYB1, suggesting that they have three exons, as with InMYB1. Although the sequence of this region in INIL11g40874 is unknown because it corresponds to the linkage of contigs, the high homology of the other parts of the sequence suggests that it has three exons as well. B: RNA-seq data of INIL00g10723 supported that InMYB1 contain three exons, not four. (TIF) [file pone.0271012.s002.tif]

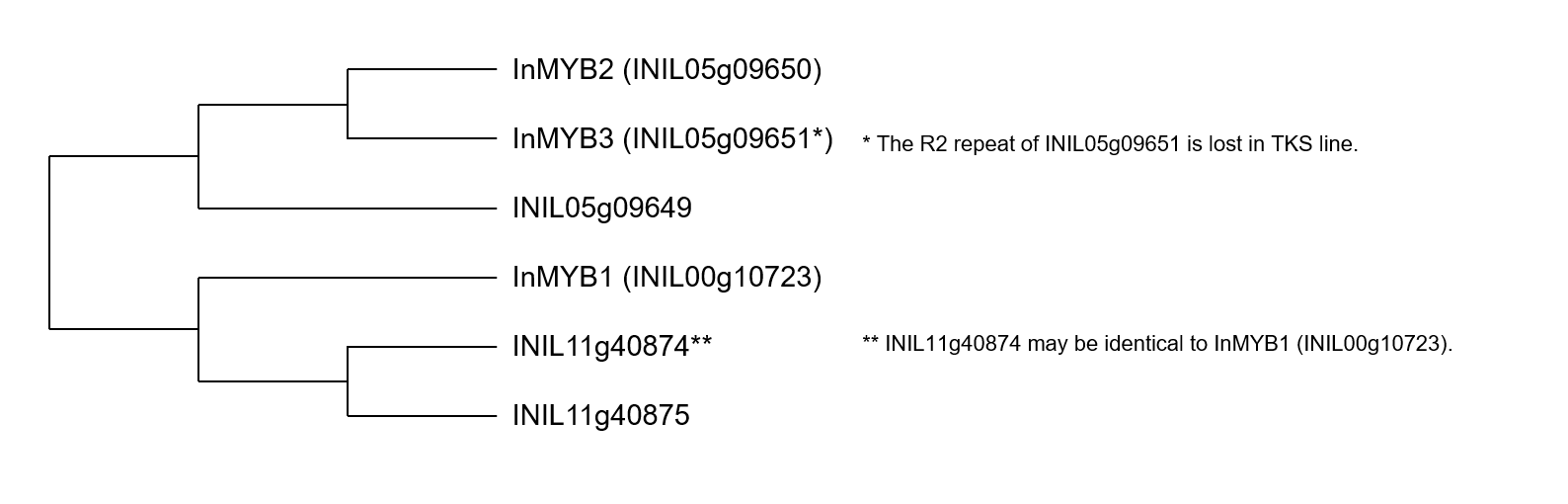

Supplement: S3 Fig — Phylogenetic tree was generated by the neighbor-joining method derived from a CLUSTAL alignment of the amino acid sequences of six members of C16. (TIF) [file pone.0271012.s003.tif]
