# Supplementary material for: Genome-wide analysis of R2R3-MYB transcription factors in Japanese morning glory
Source: PLoS One. 2022 Oct 20;17(10):e0271012. doi: 10.1371/journal.pone.0271012 (PMC9584510; doi:10.1371/journal.pone.0271012)
Supplement: S1 Table — (DOCX) [file pone.0271012.s004.docx]

**Table S2. Amino acid sequences of 126 InR2R3-MYBs.** The amino acid sequences of 126 InR2R3-MYBs are listed.

**>INIL00g10723**

MVNSSARWSPRVRKGAWSEEEDDLLRKCIQKFGEGKWHLVPFRAGLNRCRKSCRLRWLNYLHPDIKRGHFSLEEADLILRLHKLLGNRWSLIAGRIPGRTANDVKNYWHSHLKKKVVGMHMASSNSSRQDNNWDDEKGKAPQIKENILFRPRPRRFFRTSLSSPALSTLTGKAKAVAYDAPPPPHHHQLQAQPEATSPPADLLMVFNVQQNNNSMATNFPAQTTAPPSHDGVKWWDLLYDDDHQGLIDWTTDDDFPIDAEVEAGPRPEVEAGPTRRGRSAREVGRPQGRTASAGEVVRPRHRRSARPRARRMARPGRGGRPSPSRPRRGLSPGTTKARPAGHPRGSVTATPPRRRRDGVTWQRCGGAYVS*

**>INIL00g14902**

MIPHYNNSPKCLSNLSSEKVAAGASPPAKAKGKRTPCCSKVGLKRGPWTPAEDKLLTDYINKEGEGQWRTLPKMAGLLRCGKSCRLRWMNYLRPTVKRGHITPDEEDLILRLHRLLGNRWSLIAGRISGRTDNEIKNYWNTHLTKKLISQGIDPRNHKPLLVNPTNSNHPKNHNPSSSSVPIIKPTPIHVGLSNQDKTVKINSSTTVNVVGGTTNHQIDQPNSVGGTTNHQTTGNGDEEFNMDIGDDNEDNVGMDFCPDEDAFSTIFDSLMNEDVFFAAAQNNQQSNHHDITPCPQLLKIIIIISL*

**>INIL00g27132**

MDGSGGGGGYRSFSVNFPNPILPPFPPVIVHRRYNHQEILPFPERFGSSSFHGSASSTNSLLPAADAATASWLSHLQNVNFGGSRGSFPHGLVAPPPASWGNQNPEMMSAAGSPEETRREKGHDERRSSNGVAFIKGQWTEEEDRLLIRLVNEHGDRRWSVIATKIVGRAGKQCRERWHNHLRPDIKKESWSERDERLLVQVHEQLGNRWSEIAKRIPGRSENSIKNHWNATKRRQFSKRRMRSSSLQDAGSSRAGGRRRSVVLENYIKFKYFSGLSLGQPGTSLVPEQPSIQQQKLFGNSSSSSTSPTAANNGSRSNNTAEFAAFGWRTNEVDSVPEEDSWSTYLASDPNLYEMGGADQINSSTPQAPAANNTNSGAGYGSAAASHCGAPPLNRWSSSATAAAMDMDLMDLVSAASSSRCPHGYGSCCTKTPRQMF*

**>INIL00g27134**

MDGSGGGGGYRPFSVNFPNPILPPFPPVIVHQRYNHQEILPFPESFGSSSFHGGASSTNSLLPAADAATASWLSHLQNVNFGGSRGSFPHGLVAPPPASWGNQNPEMMSAAGSPEETRREKGHDERRSSNGVAFIKGQWTEEEDRLLIRLVNEHGDRRWSVIATKIVGRAGKQCRERWHNHLRPDIKKESWNEGDERLLVAVHEQLGNRWSEIAKRIPGRSENSIKNHWNATKRRLFSKRRMRSSSLQDAGSSRAGGRRSSVVLQNYIKYKYFSGLSLGQPGTAGDEQDSRDSPSILTETYDDEMNFMQKLFGNSSSSSTSPTAANNGSRSDNSAEFAAFGQRTNEVDSVPEEESWSTYLAAAASHCGTPPLNRWSSSATAAAMDMDLMDMDLVSAASSSHCPHGYGSCAAAPKPRAKCSNYYLNY*

**>INIL01g00015**

MGRQPCCDKLGVKKGPWTAEEDKKLISFIITNGQCCWRAVPKLAGLRRCGKSCRLRWTNYLRPDLKR

GLLSDAEEQLVIDLHAHLGNRWSKIASRLPGRTDNEIKNHWNTHIKKKLLKMGIDPVTHEPLNKEEITKPTSDDQSSTTTTSHAATTLPELDQNNGQQQVQVVPQSTTTQVPTEATSEEQSSSCSPTENSSSSTATTDESQAVVVLDAMCGGDDDPLLSSLLENHAPMPDAAQWDLPLPSDHQPPLIFDDNDDLEIPTLLDDNFGWLFNCQQDFGIQDFGFDCLNDPEMHIFDTVDSGNNHNN*

**>INIL01g25379**

MEEKKEAAADRIKGPWSPEEDEMLERLVEKYGPRNWSLIGNSIPGRSGKSCRLRWCNQLS

PEVEHRPFTAEEDETIIRAHAKFGNKWATIARLLSGRTDNAIKNHWNSTLKRKCGSVSSE

NLSFEPPPPRGLPLKRSSSDGLGVNMSGLHLSPSDSDFSDIYNPIARTGGGKLPLPLPPH

FPTMETPPSLSSDPVTSLSLSLPGSNEAPPLINRRNHLLPIPQLPQVSPPAKVYLPPQPP

QSFQFAAPPPPQTAEKPFFSPEFLAELQEMIRKEVRNYMSGVEHNGLCTQADEAIQNAAA

QRIGIWHRQD*

**>INIL01g25431**

MGAFHRWSSTSSTSSDSCSSGSSFSAGGNQAEKIKGPWSAEEDKILTRLVERYGARNWSM

MSKYIKGRSGKSCRLRWCNQLSPTVEHRPFSAAEDEAILAAHSRYGNRWATIARLLPGRT

DNAVKNHWNSTLKRRHQKQPQPSDLPMTAADVNVNVNSSGSTESKTEKSSETDSESPRVD

TGNRTWDDEFDPMTALSLAPPGMGRHSPPERPAESLPVVFWDAMKDVIAREVRDYITSSF

SEASTSYH*

**>INIL01g36685**

MEKETLRKGPWLEEEDEKLAAAVASLGERRWDALAKASGLRRTGKSCRLRWMNYLRSNIK

RSRITTEEELLIIELQRKWGNKWAKIAKQLPGRTDNDIKNYWRGHLRKKILREQECFKNT

SNKAQQMNTSWRSCTTADRASFKDGSLGSSVDSSEVVELPDYALVSSPYEERLWDWMSSW

SHEDSEMEHHGDGESCSRNQWCCHPRWTSDYSCIGTIWDYASSSIWDNTS*

**>INIL01g36710**

MVXAPCCDKEKVKRGPWSPEEDEKLKEYIEKNGPGGNWIALPQKAGLRRCGKSCRLRWLNYLRPNIKHGDFSDQEDRIICSLYATIGSRWSIIAAQLPGRTDNDIKNYWNTKLKKKLMGIMLSSSSSPDQKTRPNPLPPSTISSSSPFKCSNNYNYYPITDPTFPPPYASTTTFLNPSSSSSASNPVGPTIQSHHLLIISQDKEYGYGESLGLQSFGYNGVAENQQAVNNGGNPLDYSSLEEIKQLISTNNVSQNSFFVDEENKYEEKVMMYY*

**>INIL02g10399**

MGRSPCCEKEYTNKGAWTKEEDDRLIRYINKHGEGCWRTLPKAAGLLRCGKSCRLRWINYLRPDLKRGNFTEEEDELIINLHSLLGNKWSLIAARLPGRTDNEIKNYWNTHIKRKLLSRGIDPQTHRSLITSAKPTVTPQQPSPTTSSGGLSSSSSEETTYHLNLELSISLPQPADRGGAFQFRNVARGDLSVSQIRVSEWKCM*

**>INIL02g10645**

MKAKEVKAGLKRGFWTPEEDLTLKKCVETHGEGNWATISKKSGLMRSGKSCRLRWKNYLRPNIKRGMMSEDEKDLIIRMHKLLGNRWSLIAGRLPGRTDNEVKNFWNTHLNKRSRRGKRMKITPKDDDSISASIPTQSMENPNLVDGSSKQEDEMDSIMNSWMEHMGIENCNINSSISTNNLPWIFEDVPLIPILDDVLLDAFQSTGDETLLDGIHPFLL*

**>INIL02g11599**

MMGRPPCCDKANVKRGPWTPEEDAKILAYVASHGIGNWTLVPQKAGLNRCGKSCRLRWTNYLRPDLKHDNFTPEEEACILELHKTIGSRWSLIAKHLPGRTDNDVKNYWNTKLKKKLKNMGIDPLTHKPFSQVFAEFGKLSGLPSPSNQNALLKNTIKNEALFERSFPTNSRFVSAEMNQHLQIQNSPLVHNFPREPIQPPHSFTSSQYCSSSYEQPLSQFLTSSSSTPWNEFILQDPCPMADTELPRQDSKFPGTFSSDDPMTPSVQGEAGPICGFTNEGMNEDITEGLTDKSHGEASSSMAEDSFVENILARDRQMQLEYPQLLDGYFD*

**>INIL02g11823**

MGRSPCCDENTGLKKGPWTPEEDQKLINYINKNGHGSWRALPKHAGLNRCGKSCRLRWTNYLRPDIKRGKFSQEEEQTILNLHAILGNKWSAIATHLPGRTDNEIKNFWNTHLKKKLIQMGYDPMTHRPRTDIFSSLPHLIALANLKELVDQHQSSWEEQAAMARLQSEMAKFQYLHHLLSTVLGSSSATTQMDSSVLTLSNLGQAIQDSSIPFSHLPDLQPPCAAIFQQSSKDNNNNNNNMVQAQGISGFTVLSDGENSPTSPWLPSSLSPSPPPPPVVPTVDGSGASSYGGAPPSSVWPDLLLDDDPLFPDL*

**>INIL02g11914**

MGRQPCCDKVGLKKGPWTAEEDKKLITFILNNGQCCWRAVPKLAGLLRCGKSCRLRWINYLRPDLKRGLLSDFEEKMVIDLHAQLGNRWSKIASHLPGRTDNEIKNHWNTHIKKKLRKMGFDPVTHKPLSATDDDDDQKTNKTKSQTENGTSADQESPVPGTSNDQSMEEDNKSMETSTQSPVFESSIMEVSNGFCTDEVPLIEPHEILVPVSSTPTCSSSDSDNYTAGGGGDFVSSNNVNNNVNGVLEDMEMFLPSLDWQCDSIGDMGFWGDDFITTTLDSLFNNEESWKLDQLL*

**>INIL02g16681**

MGRAPCCDKTKVKRGPWSPEEDTTLKSYVQKHGTGGNWIALPQKAGLKRCGKSCRLRWLN

YLRPDIKHGGFTHEEDQIILTLYTNIGSRWSVIASHLPGRTDNDVKNYWNTKLKKKLAAA

TNNNPDVFPQTVATNFNNPTTYYGVQEMVGSSQSLHFPLPNLMEVQENCTANNVSSYNPE

VSAGYSTSFAYNNNNEKRFGTSWLGNGGIEEDVFRMDMMMSSSTSLLSSSSSPSSSPSCF

DHLMMNYGFDFQDNFTPPNILHNNFSTNHPSNGFDQY*

**>INIL02g16845**

MVRAPCCEKMGMKKGPWTPEEDQILTSFIQRYGHENWRALPRQAGLLRCGKSCRLRWINYLRPDIKRGNFSKDEEETIIQLHQTLGNRWSAIASRLPGRTDNEIKNFWNTHLKKRLQHHGSPYSPNFNVVGNITPIQIGDSSIHLRFPAAPTTVNSMYSSSPMTTKMEEAEESMQERESYQNLGTTNDDSGIVYLPSSSSVLPMELGGCETSSSIGNDAVFXIQLNKNPQKPRSVLSSRTESEHTETMAFTISPNSIHLPQSSRFSGRNSFLRASVPPPILSPPSLRFPIKCLANRLVVCAASSAAGSSSSNSDLNPYEVLGVSPIEGFDMIKASYTRKRKEAEKRGDEATAAELEMAYDKIMMSQLTKRKKGVTFGSFKVSKEIKYADKQPIVPWGPRFSKSEVKDIRINMAISAVFTAWAFIQRNGEWKPLQFLAFAFVYRIFEKLKAFESPEEGEDDGRMMRMGKRLLRSLALAFGCIAVASLGYTGLLNLIEFTGSSIPAIIYNNQELIVTTATSVLLFITASYYR*

**>INIL02g17023**

MGFVGGKGGKGKFFDRKFKKWRIPSAVSGRKRATAAGVRRSRGRFPAADDRALSASKSHSEAEKRRRDRINAQLATLRKLIPKSEKMDKAALLSSVVDHVKDLKRKATEITEALVDVPTDTDEVTIDDQHSTTAGAAINDDQASTSSSPYLKASVCCDDRPELFAEINRALKDLRLTTVQADVTSLGGRVKSVFVLQLCSDDMDAAGETSNDHMMMNSVKHSLKVALCKIAIPASTTSYRIKSKRQRVKGSWTPEEDATLVKLVEQHGPRNWSLISTGIPGRSGKSCRLRWCNQLSPAVQHRPFSPEEDAVILRAHAVHGNRWAAIARLLPGRTDNAIKNHWNSTLRRRKRSAEDAAAAAPAVDKRACLDESSAESKSEEQSKIGGAEDVLRLDGPETLLSLFPPGGKAVADPPVGENSAAEEGEEPPPSSVSEDGKEGRSKVEIEIKDKCLLIMHRMIAHEVRSYIDKLRAGGGLGPDFESNVQQRPKT*

**>INIL02g17103**

MAEVKRGAWSPEEDRKLISYIRNHGIWNWTQMPKFAGLSRTGKSCRLRWVNYLRPDVKRGPFSREEVEIIIKMYESMGNRWSAMAAQLPGRSDNEIKNFFHTHLKKHLRRQPKHAAAAADRRSKDDNTTSDDAKIIIADHCNSSSVVILESSRSCSSRVENNDAPSGGNNGTAPAFSSDTHDDDVNAFWYDVLMEAEYLNF*

**>INIL02g17162**

MGRQPCCDKLGVKKGPWTAEEDRKLISFILTNGHCCWRAVPKLAGLRRCGKSCRLRWTNYLRPDLKRGLLTQEEEKLVIDLHARLGNRWAKIAARLPGRTDNEIKNHWNTHIKKKLIKMGVDPLTHEPLQKDDDEEMLPPSSLSLHSGDDQKLKPAENDDDLEEELPKMEEHQQCSSKEAMFAAGPQVEEADYDPWMSQLLSDTFFNDLSWSFGGDECSDFAMSSSSHGSSSEWLMDLQDFGEDQSWALLPGNQPPKNMDMEIHTQDII*

**>INIL03g11384**

MGRHSSGMVKQKLRKGLWSPEEDEKLYNYVRKFGVGCWSSVAKHAGLQRCGKSCRLRWINYLRPDLKRGLFSPEEEDIILGLHEVLGNKWAQIAARLPGRTDNEIKNFWNSYHNNNSSTIIPKPTLPLNFSEFEQALRQAFDPLFLYNSEQYCHGFSTLLPSLENFENMSSSSNIMVNNNNGATFPNWETCENNNNTADYSVSAAHHQFPFSAKSEGCSWLQEGLHHRHSPDHDFSEYSLPALPQDLSGEENNLEFFMSCTKFNLWAGIVCGLKHRQRVVGIHLPSTKLNGQLSPAIGNLSFLRLLDLKNNSFTGKIPEEIGRLSRLRILSLVNNSFSGEMIWRVRFQPKESSKTRMHPKVPQSHTQKGISDETETAIAVKVLKLQVKGASKNFIAECTALRQIRHRNLVKVLTSCSSIDYHGNDFKAIVYKYMENSSLEKWLHRHENKVVDENHVPRRLSMFQRLNIAIDVACGLEYLHHGFGTPLNGFGTPLID*

**>INIL03g15019**

MASSPSKSSTSSCLTAEDMDSTELRRGPWTPEEDTLLIHYIACHGEGRWNLLAKSSGLRRTGKSCRLRWLNYLKPDVKRGNLSPQEQLLILELHSKWSKIAQFLPGRTDNEIKNYWRTRVQKQARHLKIDSNSAAFQQLIRGFWMPRLIQKIQASSIQHSDEILNTQSQQVPAPVPPDCHQTTINSGLEFSECPKTALSNDPKFEPCPNGNHQYYASHVMDGFSPSPIFPETGEFKDVIDYDLLGTGGGGGNSLIMDGFLDDSLWTMDKF*

**>INIL03g17749**

MGRAPVDKSGLKKGPWTPEEDQKLIHYIQAHGPGNWGALPKNAGLQRCGKSCRLRWSNYLRPDIKRGRFSFDEEETIIQLHSVLGNKWSAIAARLPGRTDNEIKNYWNTHIRKRLLRMGIDPVTHTPRLDLLDLSSILGSTHQLNFPSLLGLQAILNPELLRLAATNLFTSQNDNINPDLLSNPHLQNPQTMLLRKLQESQLLMNAPNFQANSDHHHQFQHPLPPCTTSDNNVSSACSDLPMQPYSTMNSSSNLQNGHVFQESLVAANGGSLLQTLENYGGGCFESNQSLSNSSNQSGLENFSFDSVLSTPLNSSSTLVNSGGANTTEDEKESFCSNLMRFDIPDSLDFEDLL*

**>INIL03g17808**

MAPDGGGMNNGGGASGRTGGARQMLKKGPWTAAEDAILMEYVKKHGEGNWNAVQRNSGLMRCGKSCRLRWANHLRPNLRKGAFSPEEERLIVELHAKLGNKWARMAAQLPGRTDNEIKNYWNTRLKRRQRAGLPIYPQEIQPQNTYHHPNQILIQQTRSSRSSSPLSALLSAPPPPTHHLPKPAPYNPPPSIYDHMFDSPAATMKSPPTTALQGHNSHFKFFSAPPGGLALTLAASSNKPNQFSSPVTPPLPKHFNAPLPIPSLPFSAALHPAMSSFSAVFAGPTGANLQERPSIQTPVPASTPTTSSGITGSLYPAPSDDANDYEVLPGLPRSNSGLLEDLLHESHALTRDDVEKINKDSSDGKGKQKSTSYEYEALLGNPTPPSVHEQAVYSFGYEGEAASAKETNSPVDDDILSLLDNFPLALPVPDWYDDGKGGGDLVNGGAFNTMQDGGGATGAVGNHKEESKSSSTTTSEATLCWNNMPCIS*

**>INIL03g18278**

MLKVKRGLWSPEEDEKLINYISTYGHGCWSSVPRLAGLQRCGKSCRLRWINYLRPDLKRGSFSPQEAALIIELHLILGNRWAQIAKYLPGRTDNEVKNFWNSSIKKKIISRACFSDHLSAIISSNIPADPNTAPFDHQTLFSLNPSYNNNVILDQAGIISAGTSSSSPSSFLQAAQMMDQNGNLVLPVMPSAPPPSDPAAAAWFLGQQPQNLEHNYSIFSAAANNDHIAPPDFDMMLFPAMPKLCEMIKAGGDAGENMPIASSSSSSSSSAVVAAAAHGGDLVFPASSLPCYPSGCNARDLQVAAGYEMEQTDTIILPSFPLPPPPSSLSPPLSLSPVSYSGQSGNQLIITYQS*

**>INIL03g21132**

MGRPPCCDKVGVKKGPWTPEEDIILVSYIQENGPGNWRAVPTNTGLLRCSKSCRLRWTNYLRPGIKRGNFTEQEEKMIIHLQALLGNRWAAIASYLPQRTDNDIKNYWNTHLKKKLKKMQGEDNANADNINGGANSSSSSHHSISKGQWERRLQTDIHLAKQALCEALSLDKSNPVQPAPNPPVQTGPYASSAENIARLLESWVKSNGPTRSNSETTTTQTGLAGSTSSPSEATFDHSIFSYNSEAFSAVESKPVFARAAAAPPTFQTQTKPNNDNIAPPHETQMPLTLLEKWLFDDAANVQPQDGIMGIPVPLPETAELF*

**>INIL04g04429**

MGRTPCCDKKGLKKGPWTPEEDEKLIDFIKKNGQGSWRSLPKLAGLLRCGKSCRLRWTNYLRPDIKRGPFSSEEEKLVIQLHGILGNRWAAIASQLPGRTDNEIKNLWNTHLKKRLLSMGIDPQTHEPSSESIGPQRRLPTTPSARHMAQWESARLEAEARLSRESKLLVPSTIGGSEADFFLRIWNSEIGEAFRKFKKGVKFACQSPISQASSSTKYGSASGTTTEMDITLAGSADAGGNPNEDPEGKCNRSYTDDPLQGSETSCSDELEDSSESALQLLLDFPTYNDMSFLGHTDTYSIYPAFLTESTLNCSSAEH*

**>INIL04g09009**

MGRYPCCKESSAELKKGPWSTEEDKKLADYIHENGIGNWQLVPKRAGLNRCGKSCRLRWTNYLRPDIKRGSFSEAEENMIIQLHKQLGNRWSKIAAKLPAGRTDNEVKNYFNTHIKKKLMRMGIDPITHKPLPNLNHLLNNLPHNYYNSFNNNNPIISPLESILRLQANLTQMANAQLLQNIAQILNNNNIPLPLVQNNINNVPLLLNNNNVTTFDNFNVGSNESPLFDHPLSTTPNSYTIESVLDQPSPMSNSFGFVPGNDDLVPRVLTSNISSLPPLVSATPETSISDQMFPMGAMQCGSGGGDDDDFSAWEKVFCDEGNSSLLESIFQ*

**>INIL04g31722**

MEGRSWCNGEKNEFGDIKKGPWKVEEDEVLLNHVNKYGPRDWSSIRSKGLLQRTGKSCRLRWVNKLRPNLKNGVKFSAEEERIVIDLQAQFGNKWARIATYLPGRTDNDVKNFWSSRQKRLARILHNSTPQSSKSWGDNNSGNSVKEVPALPDVPSLEEPKFRSSTNEESLSKHQSCSSSYIENFEAIPMLDLMNPTSFTFEPNLLQLEYAQCETKPFHGSQPQLLPFPQIPQVQTTDFSLPLEGQDFASRLGDLSFLDAFGNASGETSCEGVVVKQETDDVVINPLSPDSFIDDFSIDMFDHIEPLPSPSDW*

**>INIL04g32440**

MGRAPCCDKDGLKKGPWTPEEDQKLVDYIQKHGCGNWRTLPKNAGLQRCGKSCRLRWTNYLRPDIKRGRFSFEEEETIIQLHSILGNKWSAIAARLPGRTDNEIKNYWNTHIRKRLLRMGIDPVTHSPRLDLLDLSSILNTPSPLYNYPCQQINLSRLLGLQQPLLNPHVLRLASSLFSPPVQQQRHGLNPEFGLQNNNAGVVQENQMCNLENPGFLAAQDVPFSCPQSVTQEQTELPVQTDVTFSQMTQQPDNVDQFPPHFASFPIGNCQENEWQSNGIPSSLTEDYLPLENYGYYQPAAADPQSVMDPPPSDASTFCRSFQSVLSTPSSSPAPLNSNSTYINSCSTTTMDDEIDISYSSNLIDFDYSSILEVNEFM*

**>INIL04g32702**

MKERQRWRAEEDALLRAYVKQYGPKEWHLVSQRMNTPLNRDAKSCLERWKNYLKPGIKKGSLTEEEQRLVIHLQAKHGNKWKKIAAEVPGRTAKRLGKWWEVFKEKQQREQKENNKIVEPVEEGKYDHILETFAEKIVKERSVPGLLMASSKGGFLHADPPAPTPPNLLPPWLSNTTTSSAVRPPSPSVTLSLSPSTVPPSPVIPWLQSDRGPDKTPLTLSGFPPHHGISPPCGENPMVTELVDCCKDLEEGHRAWAAHKKEAAWRLRRVELQLESEKSCKIREKREEFESKMKALMEEQKTSLDRIESDYKEQLAGLRRDAEAKEQKLAEQWASKHLRLTKFLEQMGCPPRLAEPNGQ*

**>INIL04g34740**

MGRSPCCEKAHTNKGAWTKEEDERLIAYIRAHGEGCWRSLPKAAGLLRCGKSCRLRWINYLRPDLKRGNFTEEEDELIIKLHSLLGNKWSLIAGRLPGRTDNEIKNYWNTHIRRKLLSRGIDPTTHRPMDDPKEKVTTISFGPAQEEEDVDDEKKKMIPQDSLPTTTTVKQEYSTSPVRRERCPDLNLELRISPPYQPNNQQPPLTFTAGSAPGSLVNVDNTTVVCFACSLGIQNSKDCTCTTNGNGCYATGSATANAITSIAAGYDFLGLRNNALLDYRNLEMMN*

**>INIL05g04549**

MVGAGAKAGWRKGPWTPEEDKLLGDYVSLHGEGRWSCVARCAGLNRNGKSCRLRWVNYLRPGLKRGHITPQEEGIIIELHALWGNKWSTIARYLPGRTDNEIKNYWRTHFKKKPATGKTGSEKQDRRKKLRKINEEQENDTKPQETMTNDSSCITAATAAPMDCVCVEDNNHLGGTSANTTTTLYHQNIDDIESWVDSFAMDGLWGGELWNLDDDYCLHDPAVIQNPCGYGTDHAVNLWNGGFIF*

**>INIL05g09388**

MGRQPCCDKVGLKKGPWTTEEDNKLINFILKNGQCCWRAVPKLAGLLRCGKSCRLRWTNYLRPDLKRGLLSDHEEKMVIDLHAQLGNRWSKIASYLPGRTDNEIKNHWNTHIKKKLKKMGIDPVTHRPLCSATIDDDNKETAKQRESSVPETMDERCREVVVETPLMDQSTITDAFEEDNKNMETSPTPLDPPQVNNGFFSSPDEILATSLSSSSSSPSNSHGGFVSSVDNNNNNNNNVGSLLEDLDFLPGFDWHHDYINDVGLWVEDDDYFKELCFVDMFSH*

**>INIL05g09649**

MADSSSEPPSGVMKKGAWTEQEDNLLRKCIHKYGEGKWHLVPVRAGLNRCRKSCRLRWLNYLRPDIKRGDFNLDEIDLIMRLHKLLGNRWSLIAGRIPGRTANDVKNLWNTRLQKKTIATPSSGQEKWKDKAPKTTEKTVVIRPQPRRFVVTSSSRTLPMTGKTTIVTSEEVVQLQGHNMPPPPPPPPEAAESTSVPRLMENVDPNNSIIDPPEEAETSDNLAPWLDDFLLDMEFDGDGMTCMQEGQIEWCDFHIDSDLLDLLS*

**>INIL05g09650**

MVNSSSAWPPPSSSRLMRKGAWTEEEDNLLRKCIQKYGEGKWHLVPLRAGLNRCRKSCRLRWLNYLRPDIKRGDFSVDEVDLIMRLHRLLGNRWSLIAGRIPGRTANDVKNYWNTHIQKKVFAMARMQDNWKGKAPEIRENTVVRPRPRRFLNTSLSPTSKTGKATAVTYDAQIQGHTLPQPPEAIITTSDLVMENVQLNNTIATLPSELETTTSDDRVRWWEDLLFDKEFNDDEGNACMHEGQVGWTNLPIDMDLLELLS*

**>INIL05g09651**

MANSSAWSGVRKGLNRCRKSCRLRWLNYLRPDIKRGDFKLDEVDLIMRLHKLLGNRWSLIAGRIPGRTANDVKNYWNTHIQKKVFAMASSMQDNWKGKAPEMRENNVVRPRPRRLFLNTTSSLLSGTPPLTGKATAVTFDAQIQGHNKIPQPEATSELVTKNLQENNTIITASELETTTSNDRVQWWEDFLFDNAGSTCMNQGQVDWPNFPTDMDLSELLS*

**>INIL05g09674**

MGRAPCCDKANVKKGPWSPDEDAKLKAYIHQHGTGGNWIALPQKIGLKRCGKSCRLRWLNYLRPNIKHGEFTREEDNIICALYLSIGSRWSIIAAQLPGRTDNDIKNYWNTKLKKKLLGIKQRRDHSQEMMSSKERDKLQADQTAAMNNVCPPPVAFPAPEVLCEYPMYTTMDCITNSYSSSSFATNNYSPLVMSDVIATNDVFRPAFSFPTTALNHHDFLYGYNSPPPQQQLADRFESLSGVEMVNSSTITSASSSEGTSSNWEDISSLVYPPPKMVSTNHHLDEGCKMRDPRWDFYCSSKEYVKVKPHQTGIHVDEGVTSEKLCEKAKYSKEFPTRSDGGTTCTQSRQIRSEEENVVQFTLM*

**>INIL05g22708**

MGRSPCCEKEHTNKGAWTKEEDERLIRYIKKHGEGCWRTLPKAAGLLRCGKSCRLRWINYLRPDLKRGNFTEEEDELIINLHSLLGNKWSLIAARLPGRTDNEIKNYWNTHIKRKLLSRGIDPQTHRAVSSAQNTAAADAASPSAAVVPLALSSSAKNLDQQQHETAAAGFLPWMRNTKAENSNNTSTTTEDSNSSSGLSSEEIVLHPAAAAAPLINLELSISLPQPPAAPPSTTKLAGKEFNDQTRKNHLFFSQRAVCLCYNLGFQNSNACNCDKMMTTSSINAEAGMHSFYRPLSL*

**>INIL05g22742**

MRRPSSPTLSGSSGGRGDENGGGVKKGPWTPEEDEKLVDYIRKNGHGSWRAVPKLAGLNRCGKSCRLRWTNYLRPDIKRGKFSEEEEQLIIKLHSVLGNKWSAIAMRLPGRTDNEIKNHWNTHLRKRLLQMGIDPVTHRPRTDFNFIDALANLPQLLVAAANMGNNSNVANPLWDSINALRLCSDAAQLANELQLLQNFMALQLQLRGSVNNTTNEAQNQIPELATQFGSWNQLLDHLALLNPQLQGGLCNPGSSYNFSRLPPNISGSGSVATSSTSQNSEIQIHHPGIISNETNQRQTTNSNVSRINDDSNKLMTNAFTVSSSSSLNVPSGEDIPSNPIFPALIPASPFPENPSSSIDWETDKEKYTISANLKHDIPNHVPNATTTFEAWRDIKVDDDEATDSYWQDILYQTFSP*

**>INIL05g22908**

MGCKPAENKPKQKHKKGLWSPDEDQKLRSYILQHGHGCWSSVPINAGLKRNGKSCRLRWINYLRPGLKRGSFSLQEEETILTLHGMLGNKWSQIAQHLPGRTDNEIKNCWHSYLKKKVARMGETESQVKGESSSMGNGDSSPHSLKSSSQNSGLESFEQMEGSLTDTDQSVPEMDSSAKDTQKSIFPKILFAEWLSLDQFNNGQEFQNPSLAVPPKNTFGYNDSGFHDDFMQGLLYNEAAAYDNNSNNIEPVPGDMFQPQLKFEDQISDSGFPEFFSGGFDINDGILYI*

**>INIL05g23059**

MGRAPCCSKEGLKKGPWSTKEDLLLTNYIQQHGEGQWRSLPKKAGLLRCGKSCRLRWMNYLRPGIKRGNFSQEEEDLIVRLHSLLGNRWSLIAGRLPGRTDNEIKNYWNTHLLKKLKSSGIEPRKIVASKKKATKIVAPKKPGDAKKKPXXXXXXRKESDDNQRYKVYAPKAIRLSSGGVSRNNSVDDIAGSVSSSSGEVENKAMADGSSSSFIPWNLYELRDDFCAEVLTAAAAGDHLSPQCALPDDCLLDKVYDEYLQLLSENCFLEDDPFGANL*

**>INIL05g24002**

MGHHSCCNQQKVKRGLWSPEEDEKLIRYITTHGYGCWSEVPEKAGLQRCGKSCRLRWINYLRPDIRRGRFSPEEEKLIISLHGVVGNRWAHIASHLPGRTDNEIKNYWNSWIKKKLKKPLLSTSHTNTTTSDTTPPTTTNDHRLLQPQTSPFSFINTTNNNQLDFFNTQDSLQLRQTLFPFPNTNTNPFFPFDANAAALEGVNGDFTDDRAAAADQPFQDSPAAFWQQELQAAASFSMGMDSGYLLLPPLMENIMTLPPPPSVEVPAPPCNIALARESDHVSEWAAVDTQHQCPGSFLFWDQTDNGEELVH*

**>INIL05g24219**

MEGETEERSDLKLVAVAASDANGDGASVAVDGAEAMVLDGGGDENCGSGGKVKGPWSPEEDAILCELVSKFGARNWTLIARGIPGRSGKSCRLRWCNQLDPAVKRKPFSEEEDQIIIAAHAIHGNKWASIAKMLPGRTDNAIKNHWNSTLRRRFAATNRSTLQCGNMFDDQSVDRARASSEETKSCGYINQYKSLEGGDTSHAECRPNQTEDTPQVIEKCCAPDKTSPVIYESNLPAPAANSNPSVSRPVAKIGAFNVYYPSSAGCASSSTMPMQGPLIHPSMPDLEICNFLKSASTDPLIPSQCGHGCCRAPCGGGSSQSSLLGPEFIEFEELPPISSHKLAAIAKDLNNVAWIRSSLENPDRVPDSRTPLYASTNTEMEHSMKTDQLCFEGRNQLTVPTTNTFPKLLQFAT*

**>INIL05g28446**

MGRPPCCDKQGVKKGPWTPEEDIMLVSFVQEHGPANWRTVPANTGLRRCSKSCRLRWTNYLRPGIKRGSFTHQEEKMIIQLQALLGNKWAAIASYLPERTDNDIKNYWNTHLKKKLRLMESGGGLMIDSASRLSSSSSSSQSSSRGQWERTLQADINTAKQALTDALSLEKSHRHRPLLDFTAVPQPAPPPYASSTDNIARLLKGWMETPAKSYSSTPCISTTSNSNAAAAATPTTVTDTSSSSYCDDNAPGKDDDGICLSEAFESLFGFDSSSDQFSQSYESKPQVPLSVMLESWLLDDDNENGDLF*

**>INIL05g31792**

MELDTAELEDAAVSAGDATVESGAGGGTGEEDDEVVVLEDGSGNSDQMSDKKAAADRVKGPWSPEEDAILSRLVSNFGPRNWSLIARGISGRSGKSCRLRWCNQLDPAVKRKPFTEEEDRIILQAHAVHGNKWASIAKLLPGRTDNAIKNHWNSTLRRHYAALGKLKGEFGNMGEDVSPERSKASSEDTQSCGDVNSLKASEGKDFFSVENPNDVPSEDKNQDAVQSIEEPREPPTLFRPVARVSAFNVYNSLDGPEALFSVPRVTPLQGSSLQATSNPNIGISKLLEGAFGEQLVPCQCGHGCCGNSPQGNHNSSLLGPEFVDYSESPSFPIHELAALATDISNVAWRNSGLENGSINLFSNSASSVMCGASHAQGESLEEMRKSDHHSQLEKGKALMADPASTPISRQPLQVNTKT*

**>INIL05g32166**

MDKKPCNSQDVEIRKGPWTMEEDLILINYIANHGEGVWNSLARSAGLKRTGKSCRLRWLNYLRPDVRRGNITPEEQLLIMELHAKWGNRWSKIAKHLPGRTDNEIKNYWRTRIQKHMKPQAAGGENAATRQSSSSCCGGEQASTSQASAPDTVETYSPTSYASNNNNNNMDATFQQGPFATESNDNMWSMEDLWSMHLLNAD*

**>INIL06g15152**

MVRTPSVDQNGKRKGGWSKEEDDMLRAHVLKHGHQNWRQLPNLAGLLRCGKSCRLRWVNYLKPGIKRGNFTKDEDQVILNLHKQLGNKWSAIAARLPGRSDNEIKNHWHTCLKKFDNVGVMESSKTRSPSHADDDAQSVQSSDKSQQHLAPFGLLQAAMETLPLFSPETEPFSYFMDSAVLGSEEGTSNDGETSFQERSDQRLWNDDDSRELEQQDFFIPDRLFADEEIQSLLNDFP*

**>INIL06g15156**

MVRTPCCDKSGFKKGTWTPEEDRKLAAYVTRYGCWNWRQLPKFAGLARCGKSCRLRWMNYLRPNIKRGNYTKEEDETIVRMHEQLGNKWSAIAAHLPGRTDNEIKNHWHTSLKKNSRKPSSSSPTQNPQPKKRSSSSTRTKRQDRANSSENPIISTHEILESSQWSPQPSSSETSSSSSSSSTTTTTTTKSDGIIHDDQTVQGEFGHMECDESFWSEPFIMDNFWSNNELVVPSIDFGLLSPPSPFRDYDFLGSFDHFPEGFNNLNW*

**>INIL06g23639**

MGHRCCSKQRVKRGLWSPEEDEKLIKHITTFGHGCWSTVPKLAGLQRCGKSCRLRWINYLRPDLRRGSFTEQEERTIIDVHRIIGNRWAQIAKHLPGRTDNEVKNFWNSCIKKKLIAQGLDPNTHNLISPTQAKTTKQNNSHSHTHNHRRHQNSPPSAFTIETSSSSHRDVVPMEIKATLAAAFPPFPTPPNEKLLYHKNTIPLSHERNNNPVMDYGSCSSMEITSSSSNLSGLGILNSDNCMWGEEMGAGTETQTGGNQVFKVNTDDEFNNNGQSIEINLFDNSNIGIDFVESSLMPCAMYCNANSMDQLPWDC*

**>INIL06g37604**

MGRAPCCSKDGLKKGPWSTKEDSLLTTYIQHHGEGHWRSLPKNAGLLRCGKSCRLRWMNYLRPGIKRGNFTPEEDDLIVRLHSLIGNRWSLIAGRLPGRTDNEIKNYWNTHLLKKLKSEGFQPKPRRTSRAIPKNKKTAKPEKTGRDEKRTKRKSGSKPDAGNRHPDPPEMTTKVKVYAPKPVRVSTGTQTLPAVAAVAEKNQPPPSATPLPPLRVSTWKSRTENALAILREKDDLDGLIADQPQAAPRIVAARPTSFRRE*

**>INIL06g37606**

MGRAPCCSKVGLKKGPWSAKEDSMLTTYIQHHGVGHWRSLPKNAGLLRCGKSCRLRWMNYLRPGIKRGNFTPEEDDLIVRLHSLIGNRWSLIAGRLPGRTDNEIKNYWNTHLLKKLKSAGFESKPRKTSRTIPKKKKKTAKPDKTRSEKRKKLRHVSKPDVGNGIQDRPEMIKVYAPRPIRLSTGFARNYSFDDLATSAASSSSGNNKATLNDNGNNNNNGEELTVIPWHLYEVGDDLLDDYMDGCDLSARYSLPTSDTLMEKVYDEYLQLLSEDCCLQTCSS*

**>INIL06g37657**

MGHHCCSKQKVKRGLWSPEEDEKLIQHITSHGHGCWSAVPKLSGLQRCGKSCRLRWINYLRPDLKRGSFTEQEERTIIDVHRILGNRWAQIAKHLPGRTDNEVKNFWNSCIKKKLIAQGLDPNTHNLLSISPNQSKIHHKPKPNNNNSYHHHHQLSPSTFTIDTTKEVIPMGMKAALSSIINPPFHQPDYTNNTMMIPTSNDQLATTFDYQNPTTIMDFGSCSSSMESTTSSLIAAAAGGLGIMAGNDGAVFGTDTTFQPAQGFEPQEERQEQEQLYKVNSDEFNNGGQNMFDAASNFDFEFMDAALMPCGVYSNVNPIDQLIAWDC*

**>INIL06g38304**

MADSDRVKGPWSTEEDDLLKKLVEQHGARNWSLISKSIPGRSGKSCRLRWCNQLSPEVEHRPFTPEEDEVIAKVHAQVGNKWATIARMLNGRTDNAIKNHWNSTLKRKYSAVIGSNEGGEPRPGRILKRADSADVTVTAAPPGLRVSRDSSSDSDISYSSNDNGPAAVRSVRLALPLQAVEPRLVSEKENDPSTVLTLSLPAPGSNDGVSSLQRDQPSYRRGDSTEAAAPAPVKSESTLTLSPELLSLMQDMIRKEVETYYSALVIKDGVRNAEVKRIGLGKTN*

**>INIL07g06211**

MDGSGGSGYRPFSINFPNPILPPFPPVIVHRRYNHQEILPFPESFGSSSFHGGASSTNSRLPAADAATASWLSHLQNVNFGGSCGSLPHGLVAPPPASWGNQNRENAFAAGSLEETRREKEHDERRSSNGVAFIKGQWTEEEDRLLIRLVNEHGDRRWSVIATKIVGRAGKQCRERWHNHLRPDIKKESWNEGDERLLVAVHEQLGNRWSEIAKRIPGRSENSIKNHWNATKRRLFSKRRMRSSSLQDAGSSRAGGRRSSVVLQNYIKYKYFSGLSLGQPGSSLVPEQPSIQQQKLFGNSSSSSTSPTAANNGSRSDNSAEFAAFGRRTNEVDSVPEEESGSTYLASDPNLYEMGGADQINSSTPQAPAANNTNSGAGYGSAAASHCGTPPLNRWSSSATAAAMDMDLMDLVSAASSSHCPHGYGSCCTKTPRQMF*

**>INIL07g06212**

MDGSGGGGGYRSFSVNFPNPILPPFPPVIVHRRYNHQEILPFPERFGSSSFHGSASSTNSLLPAADAATASWLSHLQNVNFGGSRGSFPHGLVAPPPASWGNQNPEMMSAAGSPEETRREKGHDERRSSNGVAFIKGQWTEEEDRLLIRLVNEHGDRRWSVIATKIVGRAGKQCRERWHNHLRPDIKKESWSERDERLLVQVHEQLGNRWSEIAKRIPGRSENSIKNHWNATKRRQFSKRRMRSSSLQDAGSSRAGGRRRSVVLENYIKFKYFSGLSLGQPGSSLVPEQPSIQQQKLFGNSSSSSTSPTAANNGSRSNNTAEFAAFGWRTNEVDSVPEEDSWSTYLASDPNLYEMGGADQINSSTPQAPAANNTNSGAGYGSAAASHCGAPPLNRWSSSATAAAMDMDLMDLVSAASSSRCPHGYGSCCTKTPRQMF*

**>INIL07g33338**

MSMTSESEDRMTFKGSVSSPSIEEATAGGKTGGSVLLKKGPWTSAEDAILVDYVTKHGEGNWNAVQKHSGLARCGKSCRLRWANHLRPDLKKGSFTPEEEGRIIELHAKMGNKWARMAAELPGRTDNEIKNYWNTRIKRRQRAGLPIYPPDLCLKSINASKQNGDMNIFSIGDMQHPDLFQINCFEIPAVEFKNLELNQHSYPPPALVDIPASSLLDIPSSSLVGQGLKSSCGNRYFQSTVHPSKRLRGTETLFPGLSDNGSDFLPACSRFQSGNSAAAYIAAAQSFGVSSAYNQNLASDDPSLLSVIPGSHATLNGNSSYSSEPSWEKKPELPSLQSQMGSWYSPSSSPLPSLESIDTLIQSPPPTEHTESGSLSPRNSGLLDAVLYESQTLKHSKNNNTSSQQTTSDASIIRGSIVDNSCPDLHDTEWEAYGDPISPYGHSSASVFSEYTPAVNLDEAQTVVAGCKVKQEEEGMELSPMQCGDTGRDDEESFQNMFSSCFGSQNHCVLKEVFGAVLLDDFSSRDCKNMRTISMATATSSGGSPCAWDHAMSTV*

**>INIL08g00165**

MLVTRKDKRYPIRNGGRMATGKPGNNRQISPQNNNITPEGVGGQTRFGPLENLDNEGEGRDQVQQAGTMAFQQQTVVLPRIRTNYRTVRTTHEQGKDDGVQVGGHVELPRQPQNVNRGGFRSRGRGGVLTALQPNPSTLWSEGPTNGDMFPARLSIMLLITQNPLTWQGLRTHIRVIPRMWSLLTTSRRSHQMLLCKMPTQKANVKRGPWSPEEDAKLKDFIHKYGTGGNWIALPHKAGLRRCGKSCRLRWLNYLRPNIKHGEFSDEEDRVICTLYASIGSRWSIIAAQLPGRTDNDIKNYWNTKLKKKKAHFLGLMNTSPSLHPRTQTCCMIISPIPTALKAKIRVSPTSCSLVVKIIAIIYSCLVGLRPVAPLLMVSAMEANQKSSKKLMIIIITPY*

**>INIL08g04788**

MGRQPCCDKVGLKRGPWTIEEDHKLMTFILNNGIQCWRLVPKLAGLMRCGKSCRLRWINYLRPDLKRGALTEAEEEMIIKLHSQLGNRWSKIAAHFPGRTDNEIKNHWNTRIKKKLKLRGLDPTTHKPLDQPGSSIAKINGVDDHNQQQSSSSGEEEMMMIMKKDYPIPNYEYALQQTLNSSLDESLELESNIIPAGSEDMDTLSMDMYNPDDQRQEPYQTWIGSPLQWDLFNNLDGNFL*

**>INIL08g13530**

MGRTPCCEKVGLKRGRWTAEEDQILADYIHANGEGSWRSLPKNAGLLRCGKSCRLRWINYLRSDLKRGKFSPQEEEIIIKSHAILGNRWSLIAAQLPGRTDNEIKNYWNSHLSRKFYSIIRRAGSDKNIENLETELAKVAEQTKRRPGKVSRSAMKKNKTTDYKHSSNNINHAPGLQTQKQDTTIPASLYTDDPVNNNNAASSPPILMEKEDTIDTSSFLGSEHFSLDDIMPILMEEMQDPAGTILSTSSLNSAKEIERDLAKSGDSGVDIYSGLIPSSDHNQFGGENSESTATSSSFPVEHYCSLAQNIIDWDNDWQYCWDYDSGNNLCNTHNYLMPQQNDDDVMLSSPWPWDDTFYDIVVDNNNNNIAGEEGRVG*

**>INIL08g13864**

MDSVGTYLYDLGGPLALFTVGLVGVGSPRQVGDRFLWNHQQALNVENEGICPPENGVYDFPPPASLHGHAPSWPSLPEASFVDANGQTIREELRSLLNDGKDAGKMVNDHGSGSSSSLTLIKGQWSEEEDRLLIRLVKQFGMRKWSQIADNMVGRAGKQCRERWHNHLRPDIKKDGWSEEEERLLVEEHEKLGNKWAEIAKRIPGRTENAIKNHWNATKRRQNSRRKGNKNGKDAASASASSSKKKTRPTVLQDYIIAKCFNVVDNTPPSSAAAGGGISSVPEDPSIQFGSDYDDGGGGGSPSLLTHPTHDEEMNFIEALGYDIATFFPRIALRPSSRFGRMSPLPSSRYGRRPSQPSSGKKAAFFLEEGNKKAAFFSRKKASQPSFRKKAVHPSSRFGRSRWLSSRPRRRFVRRPMLPCFFQIWVKATMAWKKAIATFFMEEGKKKASQPSSRKTASQPSSRKTASQPSFKKKASQPSSRFGRSRWLSSKPRRRS*

**>INIL08g20855**

MTLKGNVNSPSVEEATGGANAGGVPLKKGPWTSAEDAILMEYVTRHGEGNWNAVQKHTGLARCGKSCRLRWANHLKPDLKKGAFTPEEEHRIIELHAKMGNKWARMAAELPGRTDNEIKNYWNTRIKRRQRAGLPIYPPDVHLQAIHESNQNEDMSTFSTGDTQHSNLLQVNYFEIPAVEFKNLELNQQLHPQSPLDIPASSLLAQGLNSSYGPRPFFSTMNPSKRLRGPDSLFTGLSSNDSDIFTAFGPYQNDTCMQVSQSSGFSSGYYQNLDPDHPSLSCVIPGSHASLNSNSSSLEPAWAKKPELPSLQSQIVSWDSPSSPLPSLESVETLIQSPPTERSESGNVSPRNSGLLEAVLYESQTLKHSKNTLPQQNSGASIIPSDIVDNTFPDFHETEWEGYSDPISPLGHSAASVFNEYTPISASSLEEHQSVEMLAGCKFTQEDAPLAPMQCDDKDDALNQIFSRPDFLLDSNCFGLKTDLN*

**>INIL08g30969**

MGRAPCCDKASVKKGPWSPEEDAKLKAYIEKQGTGNNWIALPQKIGLKRCGKSCRLRWLNYLRPNIKHGGFTEEEDNIICSLYISIGSRWSIIAAQLPGRTDNDIKNYWNTRLKKKLLGKRKQSQMNRLLLAGHHQEADNKETNGVSEENPFLQNLSNSALERLQLHMQLQGLQNPFSLYNNNNPQALWPGNKITPFQPKMADEPMNEQEQMRMLAAQPEYSSKMNELESCMNNNDGSSSLGQENNNSGEIQAAIPGFTQSEIDSLLINGGENHQISDFDYCFKQVMEGSKERLEWWNTEFDTNSWVDPPLLPPPPQQEGIMYEDYASLGYNNM*

**>INIL08g31042**

MMVSKSGAIQSLSNNNSCNEEERFGELRRGPWTLEEDTLLIKYIAAHGEGRWNALAKCAGLRRTGKSCRLRWLNYLKPDIKRGNLTPQEQILILELHSKWGNRWSKIAQHLPGRTDNEIKNYWRTRVQKQARQLKIDSNSKKFIEAVKRFWMPRTTQNRQL*

**>INIL08g31096**

MRKASCDHSHHHHEINKGAWSKQEDQKLVDYIRKHGEGGWRDLPKAAGLLRCSKSCRLRWMNHLKQTAKRGNFGDDEEDLIIKLHALLGDRWSLIAGRLPGRTDEEVENYWNSHIRKKLVDMGIDPNNHRVSCTYPRPHNSAGVAQTSAGKSRVTSPEKQRIESDGEVSDAGSSNVR*

**>INIL08g38600**

MGRSPCCEKEGLKKGPWTPDEDSQLLAYIEQYGHGSWRALPAKAGLQRCGKSCRLRWTNYLRPDIKRGNFSLQEEQSIIQLHALLGNRWSAIAAHLPKRTDNEIKNYWNTHLKKKLSKMGIDPMTHRPKMNSSFGSAANLSHMAQWETARLEAEARLVRHSKFISSSLISPHHFRLHKPSPPPPPKVPPSLDELKAWQETWTKPPRTRVSLSHVHDGAFLSNATPHQSPTTLNFSDQNLNFPDQNLNFSDHESSWNIGNPNTTGDDIIPHVAMDPLSELPTFIHGFTELSPETLTGYLDHDNIVGNCGTADVEDISRYWNSILNNLMASPVGSPVF*

**>INIL08g38603**

MGRSPCCEKVGLKKGPWTPDEDKQLLAYIEQYGHGSWRALPAKAGLQRCGKSCRLRWTNYLRPDIKRGNFSLQEEQSIIQLHALLGNRWSAIAAHLPKRTDNEIKNYWNTHLKKKLSKMGIDPMTHRPKMNSSFGSAANLSHMAQWETARLEAEARLVRHSKFISSSLISPHHFLLHKQPPPPPPKVPPSLDVLKAWQETWTKPPRTRVSLSHVHDGAFLSNATPHQSPTTLNFSDQNLNFSDQNLNFSDHESSSNIGNPNTTGDDIIPHVTMDPLSELPTFTHGFTELSPETFTGYLDDNNVVGNCGTADVEDNSRYWNSILNNLMASPVGSPVF*

**>INIL08g38605**

MGSSPCFENVGLKKRPWTPDEDQKLVAYVQQYGHGSWHALPSKAGLKRCGKSCRLRWTNYLRPDIKRGKFSRQEEQTIIQLHALLGNRWSAIAAHLPMRTDNGIKNYWNSHLKKRLSKMGIDPTTHKPKSNPFGFKEAANLRHMAQWETARLEAEARLVRRHSTMFGDDDMFVSAAALDQSPTTLNFSDQNLAAFPAVGFGDDMLLCYLEGSTCNMNTLGNPNTTGDDIIGPEMDPLSEFPTFILPENLSAHCFTGYLDSVVRNCGAADSILNLVASPVGN*

**>INIL08g38606**

MGRSPCFEKVGLKKGPWTADEDQKLVAYIQQYGHGSWHALPSKAGLKRCGKSCRLRWMNYLRPGIKRGKFSLQEEQTIIQLHALLGNRWAAIAEHLPMRTDNGIKNYWNSHLNKRLSKMGIDPTTHKPNSNPFGFKEAANLRHMAQWETARLEAEARLVRRHSTMFASSLHKPPSNPPPPPPTVPPTLDVLKVWQAAWTKPQMTARNPRVSLSPINVDDEGYMFVSAAALVQSPTTLNFSDQNLAAFPAVGFGDDMLLCYLEGSTCNMNTLGNPNTTGNDIIGPAMDPLSEFPTFILPENLGDHCSSGYLYNVVGNCSAPDSILNLVASPVGN*

**>INIL08g38607**

MGRSPCFEKVGLKKGPWTPEEDQKLVAYIQQYGHGSWRSLPSKAGLKRCGKSCRLRWTNYLRPDIKRGKFSLQEEQTIIQLHALLGNRWSAIAAHLPMRTDNEIKNYWNSHLKKRLSKMGIDPTTHKPKSNPFGFKEAANLRHMAQWETARLEAEARPVRRHSTMFGSSLHKPPSNPPPPPTVPPTLDVLKAWQAVWTEPPMTARNPRVSLSPINVDDEDYMFVSAAALDQFPTTLNFSDQNLAAFSAVGFGDDMLLCYLEGNTCNMNTLGNPNTTGDDIMGPAMDPLSEFPTFILPENLSAHCSTGYLDSVVGNCGATDSIFNLVVSPVGIN*

**>INIL08g38617**

MASSNRCIYTPPPNYSPPSSSSSSSPFGASMGMVFADMGSLSIDPKNGGTQMIQEASLPSGGEGRGSNGKEAESTGQSKLCARGHWRPAEDAKLKELVAIYGPQNWNLIAEKLEGRSGKSCRLRWFNQLDPRINRRAFSEEEEERLMAAHRLYGNKWAMIARLFPGRTDNAVKNHWHVIMARKYREQSSAYRRRKMGQLVYRRMNEEDEDQLTSTNTDGAARDHGGNSGGGGGGMRTTAIGAISSTSQSGCPFGGVGSNNNGGWMVYGGANGSPHMAASGEAIPTNNGHHHHHPPPPPFSALCAPQQPPFDPFPGQCNNQMVGIKGGGGGSNNNSTTPCDAISPTFIDFLGVGAT*

**>INIL08g38640**

MHQNMKKKSSSTNSSESTKPKERHIVSWSQEEDDILREQIRVHGTDNWTIIASKFKDKTTRQCRRRWFTYLNSDFKRGGWSPEEDMLLCEAQRIFGNRWTEIAKVVSGRTDNAVKNRFTTLCKKRAKNEALAKENSNSSVNRRVVFPSWLNSSDSISESAVPLKKQRRSHIPDPPESFSNGEKSLVSCDATNQMLRSPFAVIGQNLYSPGSNVSSHQNDEDTKDSHANGSSNETEGTFLKKDDPKILALMQQAELLSSLALKVNSENTNQSLENACKVLEDFLNHTKDGDVTKCQISEMEIQLENFKQSANELKSINECSQPSWRQPALSEESAGSSEYSTGSTLLAHGVGDNREKSEAELCALHQDIESGLQSTHIDDEFAKGISGNASNSQVDTFPACDKVNPVNETICDYSNEECCSPIQVTPMFRSLAAAIPSPKFSESERQFLLKTLGMESTSPNPSINPSHPPSCKRALLHSLWSPEEDKLLCEVKIDTE*

**>INIL09g26302**

MGRHSCMVKQKLRKGLWSPEEDEKLYNYITNFGVGCWSSVPKHAGLQRCGKSCRLRWINYLRPDLKRGMFSQEEEDLILSLHEVLGNRWAQIAAKLPGRTDNEIKNFWNSCLKKKLIKQGIDPNTHKPMAETQDNPKNSSTLPSGPNELPTFPTTPQMEPSKLPFMTTKQVFDPLFLYEPQENLITFPSACSYLNPTTYGFSSLPGLMNFDTNAQMTETDYFSDGSNSRMGSSNSSNNIGTTQMNNNNMENGSRFSWEVGNRMETLFDQYGFNNGEMIIKPEEEEEEERQLIEAHCHDYTLTSLPQDLGGPNLDVFHQL*

**>INIL09g30441**

MGRSPCCDKNGLKKGPWTPEEDLKLIHHIQLHGPGNWRTLPKNAGLQRCGKSCRLRWTNYLRPDIKRGRFSFEEEETIIQLHSVLGNKWSAIAARLPGRTDNEIKNYWNTHIRKRLLRSGIDPVTHSPRLDLLDLPSLLNLTQLNLSGLLGLQALASPEVLRLLCTLMAAQNENNSPQILLQKLQESQFLNNQVENENENENPMLLLQKLQENQSLNAPIQNHQTADFQFLDTVPSNNVCASSSLPMQPTNLSGGQVIQESLIPSNGGNLMMNSLQNNYYGVFEPEQSLSDWSNHHNFGLDSVLSTPLSSPGSTTFVNSGSTCTEDEKESDCISNIMKFEIPSASLDFEDLL*

**>INIL09g30444**

MVRGPSVDKNGLKKGAWSEEEDDKLRAYVLRYGHWNWRQLPKFAGLSRCGKSCRLRWMNYLKPGIKRGSFSNDEDEMIIKLHKELGNKWSAIAGKLPGRSDNEIKNHWHAHLKKHLQTKQDPKIIRSELKINETTAEYGSSETTQKVKIGEDESSFYDAISRYSQGTSSSEESSCLSSISISKSLDLSCNTTGWTVPEEGVMMTSSQSFGETFDCFWDDLLFADASFSPSESEGGFMSPTSQVEEEFTLPYSLFGEDDVNFLNNFM*

**>INIL09g30445**

MVKTPSVDKNGIKKGLWSKEEDNKLKTFIESHGHKNWRQLPKIAGLSRCGKSCRLRWMNYLRPGLKKGGFSVQEDEIIIGLHNKLGNKWSAIAELLPGRSDNEIKNHWHTHLKKRAKETQKPEESMELLPPTAETSEISDFEFNPQEDIFNILNPDEVSLPQNSQTVDNIPLSQEVSAASGGGISSSSSSSSISDWIIDDISTISLESFMDPLESFWTEPFVADTLLYPKNVGYPVTLFEGENFPVQTSLLGGDIVPTYFEDATWY*

**>INIL09g30446**

MVRTPCCDVSGMKKGTWTVEEDKKLAAYITKYGCWNWRQLPKYAGLSRCGKSCRLRWMNYLRPNIKRGNYSQEEDQLILKLHRQLGNKWSAIATHLPGRTDNEIKNHWHTSLKKLTEQGYASSSSPTQQPRKKPSAGRTKRSRKQQEIPSSVSAHEILESSQWSSSSSSSPSSSSSPSIGTSSPNGENTSPDQIQTPQILGSSEDESFWNEPFLLDNAFASDDFLDYRPASPFSQYGEFSSSCNLVDELVNELMDYL*

**>INIL09g33277**

MQLSSQSSTSDDDLRRGPWTEEEDGLLIHYIALHGQGRWNLLAKTAGLRRSGKSCRLRWVNYLKPDVKRGNLTLEEQLLILELHLKLGNRWSKIAEHLPGRTDNEIKNYWRTRVQKQARNLNLHSNTAAFQQLIRHFWVPTLLHKIHARSIQPQPQTTTSYQEKKPHYNSSHQPLTNSNTTTESTYFSPLSPLSDECPSINNPFHGYYGMEAFSPPLFTGSGDCFIDYDQVGEGNSSMDDFSVDSSLWSMDEIWVG*

**>INIL09g35855**

MGRPPCCDKIGIKKGPWTPEEDIILVSYIQEHGPGLLRCSKSCRLRWTNYLRPGIKRGNFTDQEEKMIVHLQALLGNRWAAIASYLPQRTDNDIKNYWNTHLKKKLMKVEGSSEDGQDGNSSSSSHQSISKGQWEKRLQTDIHMAKQALCDALSIQKPTPQPLQPVHALNQPVQPSSGSTYASSTENIARLLQTWTKPVQSRSNSETTIQSSLNNNPSLGPGSSSSPSEGTAFSSAAFDQTVFGFNSYMDENNPWVFPVESKPQTVAPQNGNTSNNFGTQLPLTFLEKWLLDDANNVPAPDDLMEMGMGIDLF*

**>INIL09g36145**

MMCSRGHWRPHEDEKLRELVKKYGPHNWNAIAEKLQGRSGKSCRLRWFNQLDPRINRSPFTEEEEERLLASHRIHGNRWAIIARLFPGRTDNAVKNHWHVIMARKCRERSKIYAKKAVLKSLNDHAASSSRQPHPGGEFTRAAFNGNLPFFDKLHYPSLPYAHSLYPLEFQGDNFVQPVTMDLDNRKKSVEFYDFLQVNTDSNKSEVIDQHARRDDEEVEQQAAAGNRSKPAGGGDVQFIDFFSTGAGRRSA*

**>INIL10g12144**

MGRAPCCDKANVKKGPWSPEEDAKLKDYIEKQGTGGNWIALPQKAGLRRCGKSCRLRWLNYLRPNIKHGEFSDEEDRVICSLYASIGSRWSIIAAQLPGRTDNDIKNYWNTKLKKKLMGLVTFSSSSSSSTTTSQKTCRPHHQTPAYPPPSIVSSSPYSSPSTISSPPPLIKCNNYFPSPTFQDFQAAAVALSIPPQHSAYVGPIQNQPHHMVMTDRVLNFGAASCSSSDGSCTVNNQITHGGKDNNQVVEFDYSETYGGLQSFVYDNGDVKPSVVESGGCYAAGNNPLDYSSLEEIKQLISTNNMCNNFFVDENKTPENVVLYY*

**>INIL10g12608**

MYINLCRHRRYYHHFPLPEMITGTNNGNGVPEIDGGGNCSRFIPRGHWKPDEDAKLKELVALHGAHNWNFIAHNLPGRSGKSCRLRWYNQLDPKLNRSTFTAEEEERLVEAQKMYGNKWATIARLYFPCRTDNAVKNHWHVLLARNERWWRQQQIGPVSAASFTSTCGGYKRMKLTNVINNPSTSSFNAAAATESTVDSKSTCSHLSLSAASFGRNPMPPDFQTPGAVTGSLSSSGVEEGKDRQCNSPRPILSTPITDIGLSITGSSSSRTEEGKGRQCYSPLPILTTPFATKIGLSITTKGSSSSSKVRRGGQSESSNSEVSGASESVANLKSTNQTTDQSNQLKKIAFYDFLGMGAT*

**>INIL10g12662**

MGRPPCCDKIGIKKGPWTPEEDIILVSYIQEHGPGNWRSVPTNTGLLRCSKSCRLRWTNYLRPGIKRGNFTPHEEGMIIHLQALLGNKWAAIASYLPQRTDNDIKNYWNTHLKKKLKKLQSPGSENNAQMGSENSPTSATSYHHHHHLVSKNSFTDRPQIATSSSSLYASSAENISRLLEGWMRASSSSSSSKGLTTATSHGGGREEEEEEDDQNRPEAAAGGGLAGSNDDNNGVIPKEEYLDSILTFGSLMNGMESGNNKQNVQEGNDNDNPPPLSFLEKWLLDESAAQVEEEEDGEDQVAVMELPTIFS*

**>INIL10g12856**

MADSGRVKGPWSKEENDLLKKLVEQHGARNWLLISDSIPGRSAMSCRTRWCNQLSPEVEHRLFTPEEDEVIVKAHAEVGNKWATIARMLNGRTDNAIKNHWNSTLKRKYNEGGEPRPWRILKRADSADVTVTAAPPPPPVLRVSRDSSSESDISYSAAAKSLRMALPLRAVEKENDPSTVLTLSLPAPGANDGVSSFQRDQPSYRRGNDQKGSRKLLRRSETHKIAFRKNL*

**>INIL10g13265**

MEDSDGGFGYNTNFRSGPPRLPLDRFLWAHHSQQQVPNVDDEMKNRSETIMFFPGNRVGEHSSSHAVASWPNPQEVIRFYEFIHGVDRSPPVGNNVNESPPGRQVKYSRSSTSTLLIRGQWSEEEDRNLIRLVNQYGVKKWAQVAEMMAGRAGKQCRERWQNHLRPDIKKDSWSEEEERVLIEVHEKLGNRWAEIAKRIPGRTENSVKNHWNATKRRQNSTRRSKKVQENKSTVLQDYIKAKYPPKLDYSGAGVSESRPSIQTDQILSPSAGEDDDDDDSSSLLMQEAYHDEDMSFMESLFGNDSTDPYPTIMAADPGNLNKHNEEPIPPPPDRYLAYLLEGATTSPASSSMEFTMNQLGSSYCYPSLSSGTKAAKDIDLMELIFPSPSQSSQGSNNNTN*

**>INIL10g16278**

MGRHSCCYKQKLRKGLWSPEEDEKLIKHITSYGHGCWSSVPKLAGLQRCGKSCRLRWINYLRPDLKRGTFSQEEENLIIELHAVLGNKWSQIAARLPGRTDNEIKNLWNSSIKKKLRQKGIDPNTHKPLSEVPSLAVMDSYPTMDNVVCSNPMSSNTPPTHEFFLNKTADLSGYLSFLNYTNAAANNIGSSFPMQHPSNSSLFLNPGNPKSNVVLPMETNSSFFENNASFSWGATKESSDLDETKWSEYLQAPFYLQQNHQAPPNHQDLFGEGKPDTQFAPQGGPCIIIVRIFRDFMLHLDSFLKFNFIENGFGNDDECAIEFLLFLICK*

**>INIL10g42763**

MEFDSNSTHVSHALQPFPMSTNYFKSEFDDVFHTDSKAVYNFQDLHPFDHHFSSSSTSPLVGPSPISFYPDLAIQMNGFDPFDPFSNDNSPASHHNNLNLFKPSQEHSEIAHNNNNGGAVVGGYLSYPNPKTLSFHHDLKPTNVVVPDESSCISANPGFRKETSGRKRSSRAHQTTDNNNNGASASMKKHSKGRKKTKSSKGQWTVEEDRLLIQLVEKHGVRKWSHIAQLLKGRIGKQCRERWHNHLRPNIKKDVWSEEEDEILIKAHVEVGNKWAEIAKRLPGRTENSIKNHWNATKRRQFSRRKCRTKWPRPSSLLQNYIKTVLNSEKNGGAGANRRNSSTNAEASTMAVVVAPPPPPPPPKAEPMEYCSGDRLVSDYDFALDEKLFGGNGIESFIEDIPGGPLELDEKYMEEMAYDMPPMMFGEPKELDLMDMISHVNL*

**>INIL11g09839**

MDKKTCCNSSSYDPEIRKGPWTMEEDLILINYIANHGEGVWNSLARSAGLKRTGKSCRLRWLNYLRPDVRRGNITPEEQLLIMELHAKWGNRWSKIAKHLPGRTDNEIKNFWRTRIQKHLKQEAATNESTVISGQGSCDKTDQVSSTSASQLSSIGQGDIYSSPQSSFVCNMEIETGFQGHHFPSHEYSNDAVWGMDDISWSMQILNGD*

**>INIL11g10021**

MGRIPCCEKENVKRGQWTPEEDHKLSSYIAQHGTRNWRLIPKHAGLQRCGKSCRLRWTNYLRPDLKHGQFSEAEEQTIVTLHSVLGNRWSVIAAQLPGRTDNDVKNHWNTKLKKKLSGMGIDPVTHKPFSHLISEIATTLAPPQVPHLAEAALGCFKDEMLHLLTKKRIGFQLHPVGPAAPVKHEDKDETIEKIKYGLSRAIKEPPVDQMLPAAGNINKPWDNHAGATSSNLGETSSGEDGSPWNQSMCTGSTCTPGEQQGRLHDKAVEDENGECSEGGKRTTTTDAPPPSIFNSDCVLWDISSEDLINPMV*

**>INIL11g10884**

MGRSPCCEKAHTNKGAWTKEEDQRLINYIRSHGEGCWRSLPKAAGLLRCGKSCRLRWINYLRPDLKRGNFTEEEDDLIIKLHSLLGNKWSLIAARLPGRTDNEIKNYWNTHIKRKLLSRGLDPQTHRPINSAAAACSGGGGGSTAKDICLDFRNAAAPAKSSNEKATLSLSQEDTKCNSGTTTEESEQQQQKDDQTALNLGLNLSIGLSTTAARPRAETPSSSNSAESVAAPPHAAAAAVMNQSVCLCWQLGWSPSGKLCTKCHNSYKWFP*

**>INIL11g16076**

MGRAPCCDENGLKKGPWTPEEDKKLSDYIEKHGHGSWRALPKLAGLNRCGKSCRLRWTNYLRPDIKRGKISQEEEHTILHLHSILGNKWSTIATHLPGRTDNEIKNFWNTHLKKKLIQMGYDPMTHRPRTDDLFSNLPNLLALATLLQPHKLEEAQAAAHNIMATKIQYLQMLFQSSPPLPMTTTTSSSSYDNNGDFWDFNLPNLSNKETDNNQTLFSIENSGTSQLLHNQLVPFNFQTHLNNDNNNKSLNSDSILPPLTDCFLNNNNNKGDSTSTSSNGDYQGTSSSSSYDWPELLLEEAFMHDIS*

**>INIL11g18427**

MRIMIKGGVWKNTEDEILKAAVMKYGKNQWARISSLLVRKSAKQCKARWYEWLDPSIKKTEWTREEDEKLLHLAKLMPTQWRTIAPIVGRTPSQCLERYEKLLDAACAKDENYEAGDDPRKLRPGEIDPNPESKPARPDPVDMDEDEKEMLSEARARLANTRGKKAKRKAREKQLEEARRLASLQKRRELKAAGIDVRHRKRKRKGIDYNAEIPFEKKPPPGFYDVTDEDRTVEQPKFPTTIEELEGERRVDKEARLRKQDIARNKIAQRQDAPSAILHANKLNDPETVRKRTKLNLPAPQISDHELEAISKFGIASDLIGSEELLEGNAATRALVANYTQTPRQGMTPLRTPQRTPANKQDAIMMEAENQRRLSQSQTPLLGGENPMLHPSDFSGVTPKKKEIQTPNPLLTPSATPGGTGLTPRIGMTPSSDGYSFGMTPKGTPMRDELHINEEMDMDGGKVGRSDSRRELRSGLQGLPNPKNEYQIVMQPLPEESEEPEEKIEEDMSERIAREKAEEEARQQALLRKRSKALQRDLPRPPAASLDLIKSSLIRADEDKSSFVPPTLIEQADELIRKELLSLLEHDNVKYPIDEKSEKEKKKGTKRKSVPVPVIEDFEEDELKEAEDLIKGEAQFLRVAMGHETESVDEFVEAHKTCSSDIMYFPTRNAYGLSSVAGNMEKLSALLSEFENVKKKMNDDTKKAQKLEQKVKVLTNGYQFRAGKFWSQIEATFKQMDTAGTELECFQVLQKQEQLAASNRINNIWEEVQKQKELERTLQKRYGDLLVEKERIEHLMDEYKKQAQMQEIEAKNRALELAKAEGDAADNKMIVAPSNEDVASVNEHESSTAVDPAQESPNKQTEDVSSMAVDPAQESPNKQTEVESSMADDTAQENPNEQTDNAQEQPSGSPKAGMDIDEVGNTTDTNDLSHTTPAARESCLTDEVHAENACNESESGIVSSGSPQLMNADDNPTSGNDGSASASAEASVSPIAEDQVS*

**>INIL11g18710**

MGRSPCCDKVGLKKGPWTPEEDQKLLAYIEQHGHGSWRALPSKAGLQRCGKSCRLRWTNYLRPDIKRGKFSLQEEQTIIQLHALLGNRWSAIATHLPKRTDNEIKNYWNTHLKKRLAKMGIDPVTHKPKNDALLSSDGQSKSAANLSHMAQWESARLEAEARLVRQSKLRSVSLQNPLEPSSPLNKPVVPPAAGSPRCLDILKAWGGGGGAGGSSGAFSVVGLGVGEPQLESPTSTLSYSENAPQISSTGAGMGEHSTAAFMKEETEDQAWKCFGNAAEHFKHGGGVEDSTAAAGFASSGLLGLTSMPAVAMESAAWTTQEHIPTGNFVERFTDLLLSANSTERSLSEAGSTESNNAGDGASGNHHYYEDNKNYWDSILNLVNSSPTNSPMF*

**>INIL11g18940**

MGRAPCCDKANVKKGPWSPEEDAKLKSYIQQHGTGGNWITLPQKIGLKRCGKSCRLRWLNYLRPNIKHGEFTEEEDNIICTLYITIGSRWSIIAAQLPGRTDNDIKNYWNTKLKKKLLGTKLQPPPPPLPFPPPADLYSLPQQTVPLPTHYSHIHNQPPLSSLSCSSTKFHCPADDLQPHEENPIIAAANTAFSSASIFSMAATNNSQGFNSFPIDLADDNLFYGYDQTQESYSYSMDLHQEMAPPNILQGPTYLI*

**>INIL11g18972**

MGEKPMDKPKQKHKKGLWSPDEDQKLRNHILKHGHGCWSSVPINAGLQRNGKSCRLRWINYLRPGLKRGTFSLQEEEAILALHGILGNKWSQIAQHLPGRTDNEIKNFWHSYLKKKVSKMAEAERSMENGECSVHSVKSTSQSSGLDSFGQMEGSLADTDESIPNNLDFPKTAQKSNLPKVLFAEWLSLDQFNGQDNFQTANHAVLPRNNFGYNGSELQDPFMHGLLLNEETYGNGIQPVLNNATVDDMFQPELKFEDQITVSGFAEFFSGGFNVSSNDIMYDELYLRRNFREE*

**>INIL11g18974**

MGRTPCCNKEGLRKGAWTAEEDKILVAYITKNGHGNWRSLPKLAGLLRCGKSCRLRWTNYLRPGIKRGQFSSEEVDAIIQLHTVLGNKWSVIASHLPGRTDNDIKNFWNSHLRKQRSDPNHQNHPIPHPHGNIDEKVQWESVSVAPSLLDLPPASKMDRDPFLRLWNSEVGEAFCGFKKPFGVPCQSPVSSSSKFESSSGITLHSQPAASKLLSSADTVEEEEEEDTKSYELIDPSETTLKILLDFPPVVNDMGFFQGPGDNLSIYLQN*

**>INIL11g40874**

MVNSSARWSPRVRKGAWSEEEDDLLRKCIQKFGEGKWHLVPFRAGLNRCRKSCRLRWLNY

LHPDIKRGHFSLEEADLILRLHKLLGNRWSLIAGRIPGRTANDVKNYCTAILRRRSAREV

GRPQGRTASAGEVGATSAPEVGATSAPEDGATRQRGTTVAEADPGVA*

**>INIL11g40875**

MPLVPTRRKLKAAALKPIPHVRHQKMFPFSGISEVEGHDGNQVKANLPVVPSTKADSNIGVATATQRKATASSQQAKQIIPLNPPPLKKHGCPSAVFYCTCRSFCFHFFCILALASYPLHAKQRKYPLRKNLRQPAMVNSSARWSPRVRKGAWSEEEDDLLRKCIQKFGEGKWHLVPFRAGLNRCRKSCRLRWLNYLHPDIKRGHFSLEEADLILRLHKLLGNRWSLIAGRIPGRTANDVKNYWHSHLKKKVVGMHMASSNSSRQDNNWDDEKGKAPQIKENILFRPRPRRFFRTSLSSPALSTLTGKAKAVAYDAPPPPHHHQLQAQPEATSPPADLLMVFNVQQNNNSMATNFPAQTTAPPSHDGVKWWDLLYDDDHQGLIDWTTDDDFPIDVRIATAKDVAICLKKTKLPRHLGITANCSEANGLIRRHARSADPRVGTASAGEVGATSAPEVGATSAPEDGATPAEGDDRRRGRPRRGLSPGDHEGAACRPPPWQRHGDATAPAP*

**>INIL12g01151**

MRKPDPMGKMGNNNNKAKLRKGLWSPEEDEKLMSYMLRNGQGCWSDIARNAGLQRCGKSCRLRWINYLRPDLKRGAFSPQEEELIIHLHNILGNRWSQIAARLPGRTDNEIKNFWNSTIKKRLKNNANNQNNSSSLSPNTSDSSSENPRAMIGGATASALILPAMHHHHQPEFMASICMDSSPSSSSNMLPHFNPFPPQQPFEEAAAAGLFGLPPCLAAQLVGVGGPSAAGECGFLGDYNNVVVEPCYGKANAVNSNNNHLYEEGSIKVEDYMVNHHWSGESLRIGELDWEGLLANVPSLPYLDFQVQ*

**>INIL12g01339**

MKGRRSSYTCSYTHHHHHHHHSNGDSGELTEMKKGPWKVEEDEVLLNHVKKYGPRDWSSIQSKGLLQRSGKSCRLRWVNKLRPNLKNGVKFSAEEERVVIDLQAEFGNKWARIATYLPGRTDNDVKNFWSSRRKRLARILHNNNHNSAASSSQSNMSPEKNSVAVEEPPAFHQASSLEAQKLSSIDDEQEGMLVSKPQSCSSSSNVVGKSEEACNVVPAMPPENIPNPFIFEPVNLFHPHLLSFPEFPQLLTDDGIALPPAIESLPQDGFNDNLGPLWDPSLLDMFGSSGACDASGMGNGMPQLPFPSPWGAPEGSFRREDGVKKEAGTLTPDSFIDDFPIDMFDHIEPLPSPSDW*

**>INIL12g01471**

MGRAPCCDKKGLKKGPWTPEEDEKLTEYIKKNGHGSWRSLPKFAGLLRCGKSCRLRWTNYLRPDIKRGPFSPEEEELVVQLHGLLGNRWAAIASQLPGRTDNEIKNLWNTHLKKRMISMCIDPQTHKPSSDANGLMWTMPTTPSARHMAQWESARLEAEARLSRESLHLVPSPTTVGSETDFFLRMWNSEIGESFRKLKKGEKADCQSPISETSSSMKCGSASGTTEVDPTLTVHSAADCNLNKDAEQKSCKSYIEYPLSEMSCTDELDDSSESALQLLLDFPTHTDMSFLEHTDAYTIHPTPFSPKIL*

**>INIL12g03255**

MGRAPCCEKTAVKRGAWSKEEDQILINHINKYGHGNWRNIPKNAGLVRCGKSCRLRWVNYLRPDIKRGHFTSEEEFLIVKLHKIFGNRWALIAAKLPGRTDNEIKNIWHTRLKKRLHEFDIPADNQVAGDTLKNDSATNFEGAAPSPPAEDNDPATNNILPPHSKSSSGAAGAAAAAPNNPSDGGSAGDDDQFWTQINDKSGFEFNGADFNVGGVDPGSLQFWQDHLLTWNDDEFLDLWN*

**>INIL12g03514**

MFFPENRFFDVSSPCFSPLIIGEHSSSHAASLAGGAPSWQNPQEVIFYDAATFVHGVDRSAPVIGNNVNESPGRLLNSRSSSSTLLIKGQWSEEEDRNLIRLVKQYGVRKWAQVAENMSGRAGKQCRERWQNHLRPDIKKDSWSEEEERVLIEAHEELGNRWAEIAKRIPGRTENSIKNHWNATKRRQNSRRRSSKKVQKNNNNDSALNKSTVLQDYIKAKYPKVDSGSISGAAVLSESPSIQTDQILSPPSPAGEDDGSSSLLMQAEAYHDEDMSFMESLFGNNSTPYPTILAADPDNLNKHNEEPILPPPDRYIAYLLEGATSSSSMEFTMNQLGSSYCYPSLSSGSKAKDIDLMELIFSSPSQSSQGSNNNTN*

**>INIL12g08387**

MVRAPCCDKIGVKKGPWSPEEDQILISYIQRNGHGNWRALPKQAGLLRCGKSCRLRWINYLSPEIKRGNFTKEEEDAIIHLHETLGNRWSAIAARLPGRTDNEIKNVWHTHLKKRLSNYEPTKKHPTKTTTATSEKHSDIQHGTGPTSPHQTSSSDDMSSSSSATDSSAITEDPAMIKLENADSAEGFVEIDESFWTEVPSFGSTVSSLENFMEQSGGDMNMNMMVEDDRDFWYNLFVRAGELTELLEF*

**>INIL12g21835**

MKTEIEDKMTSKGGISDDTTGGGNAGSVGGKTLKKGPWTSAEDAILVEYVTKHGEGNWNAVQKHSGLARCGKSCRLRWANHLRPDLKKGAFTPEEESRIIELHAKMGNKWARMAAELPGRTDNEIKNYWNTRIKRRQRAGLPIYPPDICLKALNESKQNEDFSTFPNGGTHHPDLLQINNFEIPAVEFKSLEVNQQLYPPALLDIPPSSLLDIPASSLLAHGLNSSYGNRSVLSTMHPSKRIRGSEPLFPGLNANAGDILPACSPYQNDSSVEIAESFVFSSAYDHSLASSHPSFSGVISGSHASLNGNSSSLEPTWAKKLELPSLQSQMGSWGSPSSPLPSLESVDTLIQSPPTEHNTGSGSLSPRNSGLLDAVLYESQTLKHTKSNSSEQTSGAFMMPGDIMDSSCPDLDGTELEAYGDPISPLGHSVASVFSEYTPISGSSLDEPQSVETMPGDEAKQEDAGSVSVQCDDKNDASNHEEMFSRPDFLLDSNCFDMKADHGKNHSILKDAFGAVLFDDFSKDCKKITGLGCVQDSCAWDAMSTF*

**>INIL12g22053**

MGRAPCCDKSKVKKGPWSPEEDAKLKDFIDKNGTGGNWIALPQKAGLKRCGKSCRLRWLNYLRPNIKHGEFSEEEDRIICSLYATIGSRWSIIAAQLPGRTDNDIKNYWNTKLKKKLMAMMLLPPSSSSSSSSSKHRSIISSSSMSPLFSAAPTGPSVLLPTPPQQQQFSFYTPHRSFSGLESPSQLHHPDFGSSPYSSFNFQMSHQDQQASLLNPALMQQYHHHHHPAAMNKGNHHNLLMFGGSDQVSGSSSSDGSCSQISYERSKITDHNVKQEPADLGKYSFQGDPHHQIISSTNAGFEDTNNQIQGHMFFLDYGNGPPQGAG*

**>INIL12g24707**

MRTQSSEKGAAAGEAPSKAKGKRTPCCSKVGLKRGPWTPAEDKLLTDYINKEGEGQWRTLPKMAGLLRCGKSCRLRWMNYLRPTVKRGHITPDEEDLILRLHGLLGNRWSLIAGRIPGRTDNEIKNYWNTHLSKKLISQGIDPRNHKPLPVNPTNSNHPKNHTPSSSSVPIIKPTPIHVGLSNQDKNVKMNSSTTVNVVGGTTNLQNDQIDQPNSVGGTTENHQPTGNGDEEFNMDLGDDNEDNVGMDFCPDEDVFSTEHRSSLYGSCSVIMEDDFSNLLA*

**>INIL12g24714**

MVNPSGERPPNPPRSGDGRDTGHEVSEEAPASVRAEDGGRGGGERAKVIHSEQPPVGPIVVPRGRGPRRADAREVGLEEAEGESAYRPPVPATRASPPAGCIDKDEVLAVIPPPDEWMQEMIRYKEQDGWEAASCVEVVAGWEGKCGDKLSGGAAGASPPAKAKGKQTPCCSKVGLKRGPWTPAEDKLLTDYINKEGEGQWRTLPKMAGLLRCGKSCRLRWMNYLRPTVKRGHITPDEEDLILRLHRLLGNRWSLIAGRIPGRTDNEIKNYWNTHLTKKLISQGIDPRNHKPLLINPTNNHPKSHTSSSSVPIIKPTPIHVGLSNQDKTVKMNSSTTVNVVGGTANHQNDQIDQPNSVGGTTTDHQTTGNGDEGFNMDIGDDNEDNVGMDFCPDEDAFSTFFDSLMNEDVFFAAAQNNQQSNHHDITPLPSTSENNNNNQPLNPFQHMNFPFSTEGWVDDDDFLP*

**>INIL13g07867**

MDHVKGGGGGAYKNVVQQQSEDDADLRRGPWTVEEDFTLINYIAHHGEGRWNSLARCAGLKRTGKSCRLRWLNYLRPDVRRGNITLEEQLMILELHSRWGNRWSKIAQHLPGRTDNEIKNYWRTRVQKHAKQLKCDAAAGVQHPNNAAPAIPDYNAAVNFPSDNSSTAASSDSFATQVSPVSDLTDCCYNFHQVNQSATHDYYQPNNNHLIGGYGDSLTSPTAFFNQNLDFQMMENNNNNNNHQWIDGGADVVSDNLWNIEDMWFFQQQFNNNSHP*

**>INIL13g07908**

MGRAPCCEKEGLNRGRWTKEEDERLINYIHQNGEGSWRSLPKNAGLQRCGKSCRLRWINYLRSDLKRGNFTADEEETIVKLHTSLGNRWSLIASQLPGRTDNEIKNYWNSHLSRKIYSFRLSINAPMAVQVAEDAAAARRRRGGRVSRSVAKKYNTASFHVATASFRNKHKPPGSQGSSSAVHNSDGGTAINASEGSGKDGKAFAPKGRAENDVFSCDNRQLDCQVFDEMTREEDASGALLAINGGRGRGHPSESGESEGYSDGQTAATTTLGMYTYQLPAPGDSIFDDDDWVNWSLGDDAFQGYDEFWDGLDDMFAWPWDDNTNTNTANGS*

**>INIL13g08188**

MGLKRGPWTPEEDQILISYIQKNGHGNWRALPKQAGLLRCGKSCRLRWTNYLRPDIKRGNFTKEEEDTIIQLHEMLGNRWSAIAARLPGRTDNEIKNVWHTHLKKKLKGYQPPQNAKRHLSGKAAGAGDGGAASSTTSEDDGMNVAVSSPERSSSTSTSSEMSSVTGGVAAVDAATAGVKQEDVNSSPEYVPEIDESFWTEEAAAGLPWVQVDEFPVGASLANSEDVDRMMWHTRTEDDDMDFWYNVFVRSAGELPELPEF*

**>INIL13g08245**

MMGTEQMGWGIMAGWRKGPWTAEEDRLLVEYVRFHGEGRWNSVARFAGLKRNGKSCRLRWVNYLRPDLKRGQITPHEERIIVDLHARWGNRWSTIARSLPGRTDNEIKNYWRTHFKKKAAANSSDHPKPRILRQQQQQQKKKQEESNHQIDMRKMMSSFEEIDENRLSSVPQPQIAAGLLSDQEQQGFLSSMIHGCAEVSSSTEDIIMCDGLWNLDDFHGSFSPMASANKACFLQPVAAPFY*

**>INIL13g15530**

MGRHPCCKDSEEVKKGPWTAEEDQKLSDYVLKNGHGNWQMLPKKAGLNRCGKSCRLRWTNYLRPDIKRGDFSQEEEQIIINLHSSLGNKWSRIAAHLPGRTDNEIKNFWNTNLRKKLLRMGIDPKTHQPITDINLLLNLSHQMLSNTNNPLGSALRLQNIFQFLNSYPILFPTNKQENFPMGLDNNNTIDTINNYVSATHNSSDFIGSDFNPHMKILTDAGENNGHSEYSLPSLVQSSSEYIPTLDQTIIPSPGGLEANNNEFLAWEEFLKNEEDCSSLWNDILQAKQT*

**>INIL13g40955**

MTSRKSGGARMHVHKGAWTAEEDKKLTHYIENHGAKKWKTVAIKSGLNRCGKSCRLRWLNYLRPNIKRGNIAEDEADLILRLHKLLGNRWSLIAGRLPGRTDNEIKNYWNTHLSKKVTQLGKSSLPATENQPPKNGMADAEQMGGNKESEEDPELNFDVDEFFDFSVEGTYGTEWVNKFLEVEKGMP*

**>INIL14g04070**

MGRTPCCSDKDGLKRGPWTAEEDQKLIDYINKHGYGNWRTLPTNAGLQRCGKSCRLRWMNYLRPDIKRGRFSLEEEQIIIQLHSIIGNKWSAIAARLPGRTDNEIKNYWNTHIRKKLLRMGIDPVTHRRRVDLLDLSSILNNNPSLLYNSRILGAQTFANPHLLRLAASQHHNNNVINSDTANNVVQDTQQLHAPPPPPLVQDFPVYSPAMAAAQLTQQPNGEFGLENCPANDFWLGTGLPESELTQDYLLPPLQNYGYYEPAVDPQSAMDPPAPAADESYRFGFRQGWSTPSSSQVNSSSTTTTEDEREISYCSNLWNFDVANIF*

**>INIL14g06864**

MGRHSCCYKQKLRKGLWSPEEDEKLINHIAKSGHGCWSSVPKLAGLERCGKSCRLRWINYLRPDLKRGTFSQEEENLIIELHALLGNKWSQIAARLPGRTDNEIKNLWNSSIKKKLRQKGIDPNTHKPLPQVENAEKPQVFNNLVEFNNPKSSQMISGFNPNPNSVLDHHCSSSVKLQGSPAFFNTSGFPWLVSGSEKSEKLSDPEDIKWSEYLLGNAIPNQQFTVTEEGSFSTTASTWLQNQHPSLQAANLCTGSKPFQRLSAASSQFS*

**>INIL14g35326**

MSFKAFESSSSGAFRFPPPPDLRFPPLGAALGLEEPEKRSADQTHRLCARGHWRPHEDGRLRELVAKHGPQNWNLIAEKIPGRSGKSCRLRWFNQLDPRINTKPFTEEEEERLRAAHRMYGNKWAIIARLFPGRTDNAVKNHWHVIMARLHRHQTTGGARRRQPRNHNNNNTMDSNNLNDESAASTCTDLSLSTSSSSALYMAGSSLKGVKAAEASGRRPESSPDSVANNNTTGSEAEMCGQNQTAIYDNNKMLFFDFLGVGAN*

**>INIL14g35341**

MGRSPCCEKAHTNKGAWTKEEDERLIAYIKAHGEGCWRSLPKAAGLLRCGKSCRLRWINYLRPDLKRGNFTHEEDELIIKLHSLLGNKWSLIAGRLPGRTDNEIKNYWNTHIRRKLLSRGIDPTTHRPINGGAAEAKETTATTISFGAVKPEDAENYSITTGKDLGPKKEEKEEETLLFKSEEPQVVEACPDLNLELRISPPSFQETQPPLPLEAAGSGGGGRVNGLCFACILGIPNSIDCTCNNNEDYSSSN*

**>INIL14g41452**

MEAFNRSSSTSSTSSDSSSSESSFSAGGKAPRDGNRPERIKGPWSAEEDKILSRLVERYGPRNWSLISKYIKGRSGKSCRLRWCNQLSPSVEHRSFSPAEDETILAAHAKYGNRWATIARLLPGRTDNAVKNHWNSTLKRRYQHQNQNQNQNQNQTFDFTEVADAKSSPSGSVFFGLENSTLCMNINAGESPRVNTSSGRTFPLSHCDDEYDPMTTLSLAPPGMGGHELPERRTESLPAGFWDVMREVIAKEVREYVTSSFSGPSTGFH*

**>INIL14g41510**

MATSARRKVTDRIKGPWSPEEDELLQKLVEKYGARNWSLISKSICGRSGKSCRLRWCNQLSPQVEHRAFTAEEDEIIIRSHSKFGNKWATIARLLHGRTDNAIKNHWNSTLKRKCVSKAKSFVKPPQQPQPVKGSSGLFSAPDSPPGSDLSSSSLSGGVPPHVYRPVARAAGVVPPPETVSSATNPITSLSLCVPGSDCSEIPHSVSQPSPAPVSLPQMAPPVSSFLPQTYGSFQFGSPPTAEKRLFSPEFLAMLQDVIRKEVRDYMSGIEHGRLCFQTEAVQNAIVNRIGITKIDG*

**>INIL14g41566**

MGRAPCCDKNNVKKGPWSPEEDAKLKAYIEEHGTGGNWIALPQKIGLKRCGKSCRLRWLNYLRPNIKHGGFSEEEDNIICSLFISIGSRWSIIAAQLPGRTDNDIKNYWNTRLKKKLFGKQRREHGLKAGNGNVARQKQAEMRKAAVAAAARENPMMMMMAPLGNTNNSPPWPELPVLDPIRYPAADNEPGFNDHHSIRNLLIKLGGKFRDDNDDDLKHKNLAAYPMENPSPVLVPPPPPPLYQLSSAPTTDTTLNSPFSINEYNMEAELRSLPGENCFPEQIPYTDNTNIPQKMDGLEFLYDNMLNNNGRLGSSSGGAMMDWSEMMSYCSLAFAPPLSTYTTTAVQPANGLPAAQPHSAALFDGEELISYSGTPPQ*

**>INIL14g41878**

MVETTGGGGGGASASSDDSSRTCPRGHWRPAEDEKLRQLVEQYGPQNWNSIAEKLQGRSGKSCRLRWFNQLDPRINRRPFSEEEEERLVAAHRIHGNKWALISRLFPGRTDNAVKNHWHVLMARRQREQSKLSVKRTYQQVFNNVNVNDTNVFRRRYKSPDPSNKIISFFEFQNNPDSSRPTFSLSKYTPPAPAPYSSPPLRPPSSDYSLLRHHKNSTNKNLGFSLVGSHGGVIRSDPPNLKPWNFLNYSSTFNDGAKNHERENDFNDI*

**>INIL15g14256**

MGRSPCCEKMGLKKGPWTKEEDQILVDYISRHGHGNWRALPKRAGLLRCGKSCRLRWINYLRPDIKRGNFSHEEEDAIIKLHQALGNRWSVIAARLPGRTDNEIKNIWHTRLKKRLNDYGLVPPQPSLKSKSQPLKPFAMDLQTPSSPPHSSTTTSTDVHASSYCSISSHCVVSDAVLLQSDLPEVDESFWSQVFSSENSSDAGDLPATVDGESRFDSTKNETYETNSSVEFWHRLFSKTENLPVLPEL*

**>INIL15g23810**

MYKRHIVSWSQEEDDILREQIRLHGTENWTIIASKFKDKTTRQCRRRWFTYLNSDFKKGGWSPEEDVLLCEAQRIFGNRWTEIAKVVSGRTDNAVKNRFTTLCKKRAKNEALAKENNNSFINLNNKRVIFPGAGSPFKKLRRSHISDLPENCNSGEKSLVTCNAVNQLQRSPFSVLPNVGSSLPTNQNVSIKDSPTNGTFLKKDDPKILALMQQAELLSSLALKVNSENSHQSLENAWKVLQDFLNQKEDDVLKGRISEMDFKGLAEDLKNINVPSQSWRQPALSEDSAGSSEYSSESTLLSHAPGDKKEQCEAEVSALHQDIESGLQSTEIGDRTTINAFSGGTPANATCTEDILPTCDKVGANEEAAAEECSSPLHVTPLFRTLAAAIPSPKFSESERQFLLKTLGMDCTSPKSSINNTSHPPSCKRVLLHSL*

**>INIL15g27998**

MATMSQKKDADRIRGPWSPEEDELLQRLVEKHGPRNWSLISKSIPGRSGKSCRLRWCNQLSPQVEHRAFTAEEDEKIVRAHAKFGNKWATIARLLSGRTDNAIKNHWNSTLKRKSAAAAVSEDSSGFEPRRKRSSSVGAAGVNVSGSGSVSGFYVSPGSPSGSDLSDSSLPHLYRPIARTGGVFPVTQIETASSPAANDPPTSLTLSLPGSDTSETPSPTSVTPTSQLVHSPHAPHAPPPPPPPPPLPRTPISFTPVVAPPQPPPPPPPPPLPSLPPQTHQNFEFLPTPPPQMAEKPLFSPEFLAVLQDMIRKEVRTYMSGVDKNGLCMPTEAIRNAVVKRISLSRIE*

**>INIL15g29270**

MASISRKDMDRVKGPWSPEEDELLQQLVQKHGPRNWSLISKSIPGRSGKSCRLRWCNQLSPQVEHRAFTPEEDDTIIRAHARFGNKWATIARLLAGRTDNAIKNHWNSTLKRKCSSMSADEGNDLADRLQHQPLKRSVSAGAAVTLHLNPGSPSGSDVSESSLPVMSPSHVFKPIARTGGVLPPPVETPPPPPLNDPPTSLSLSLPGVDSSSDVSPRLTESTQPISPIQLFSSAMHTPPPPPPPPLPLPVPFQQPLEKFDLGGGAPPPMACPIPPKEAVPAQQDRVFLPFSQELLAVMQDMIKTENGIGRGMCMQPATSNHGLRYAAAAAAAATVNRVGVNRLE*

**>INIL15g31180**

MGRQPCCDKLGVKKGPWTAEEDKKLITFILSNGQCCWRALPKLAGLRRCGKSCRLRWTNYLRPDLKRGLLSQDEEQLVIDLHASLGNRWSKIASKLPGRTDNEIKNHWNTHIKKKLIKMGIDPVTHEPLIKDTTDSTSDKGNGQLQVQVVPEGTPPTVPNLISEDLNSSCSTSENSSITSTNDEFQRVLDTMSDNDPLLSSLLENNAPPVDLTWNLSDDHQMIFENLTIPKLDENFAWLMDHQDFGIHDFGYECSNVATLM*

**>INIL15g31267**

MGRAPCCSKEGLRKGPWSAKEDLLLTNHIQHHGEGQWRSLPKKAGLLRCGKSCRLRWMNYLRPGIKRGNFSQEEEDLIVRLHSLLGNRWSLIAGRLPGRTDNEIKNYWNTHLLKKLKSAGIEPKPRHSKDSKKKPAKPRPNPQKLTANTKKKHKKARNDEQSPRQDSDQTATAPEKRTKVYAPKPIRLSPSPAFSRNHSLEDVAGSVSSSSGEVDNKAVVLQGTTAEPPPPPFIPWHLYELGGDVDFCDQILDGCDLSSPKCSGPTSDGLLEKVYDEYLHLLSENCFEPLTDDCLCDYPFVDYNVAPTSSNNSSLN*
